# Causal Effects of Early Discontinuation of Proton Pump Inhibitors on Kidney Failure

**DOI:** 10.1016/j.ekir.2026.106709

**Published:** 2026-07-20

**Authors:** Yuki Kawano, Tatsufumi Oka, Yusuke Sakaguchi, Hikaru Kadono, Ryuta Uwatoko, Shungo Fukuda, Yohei Doi, Nobuhiro Hashimoto, Yasuo Kusunoki, Satoko Yamamoto, Masafumi Yamato, Ryohei Yamamoto, Isao Matsui, Masayuki Mizui, Jun-Ya Kaimori, Yoshitaka Isaka

**Affiliations:** 1 Department of Nephrology, The University of Osaka Graduate School of Medicine, Suita, Japan; 2 Department of Kidney Disease and Hypertension, Osaka General Medical Center, Osaka, Japan; 3 Department of Nephrology, Toyonaka Municipal Hospital, Toyonaka, Japan; 4 Department of Nephrology, Ikeda City Hospital, Ikeda, Japan; 5 Department of Nephrology, Sakai City Medical Center, Sakai, Japan; 6 Health and Counseling Center, The University of Osaka, Toyonaka, Japan

**Keywords:** chronic kidney disease, kidney failure with replacement therapy, proton pump inhibitor

## Abstract

**Introduction:**

Proton pump inhibitors (PPIs) are associated with adverse kidney outcomes. In clinical practice, however, long-term PPI use without a clear indication is common. It remains unclear whether discontinuing PPIs reduces the risk of kidney failure in patients who initiate PPIs. Using a target trial emulation approach, we estimated the causal effects of initiation and discontinuation of PPIs on the risk of kidney failure.

**Methods:**

Using data from the Osaka Consortium for Kidney Disease Research, a retrospective multicenter cohort in Japan, we emulated 2 target trials in patients with nondialyzed chronic kidney disease (CKD). First, we compared the risk of kidney failure with replacement therapy (KFRT) between PPI use and histamine_2_ receptor antagonist (H_2_ blocker) use, using an active comparator new user design. Second, the estimated effect of PPI discontinuation within 90 days after initiation on KFRT was evaluated using a clone-censor-weighting approach.

**Results:**

We identified 3512 PPI new users (76.1%) and 1102 H_2_ blocker new users (23.9%). At treatment initiation, the mean age and estimated glomerular filtration rate (eGFR) were 71 years and 30 ml/min per 1.73 m^2^, respectively. Over a median follow-up of 2.1 years, 654 (14.2%) progressed to KFRT. PPI initiation was significantly associated with a higher hazard of KFRT (hazard ratio [HR]: 1.39; 95% confidence interval [CI]: 1.13–1.72). Of 4187 patients who initiated PPIs, 1254 (30.0%) discontinued PPIs within 90 days. Discontinuation was significantly associated with a lower hazard of KFRT (HR: 0.85; 95% CI: 0.72–0.97) than continuation.

**Conclusion:**

Discontinuing PPIs within 90 days after initiation was associated with better kidney outcome.

## Graphical abstract

**Figure:**
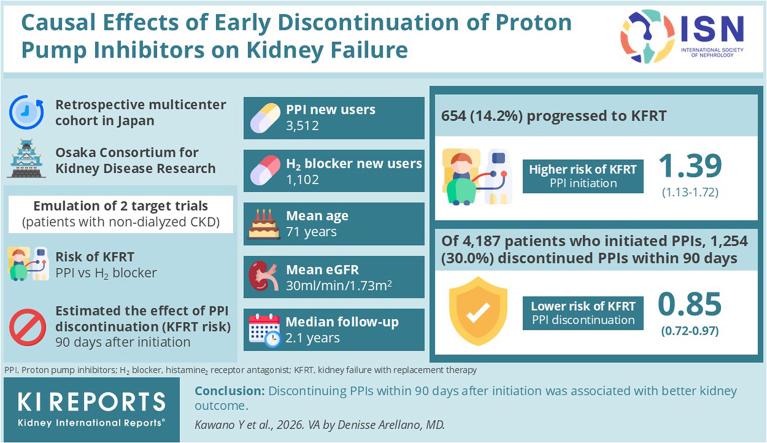

PPIs, potent gastric acid suppressants, are among the most widely used medications worldwide, particularly in patients with CKD.^1^^,^^2^ With the marked expansion of PPI use, inappropriate long-term prescriptions without clear clinical indications have also increased.^3^^,^^4^ Overprescription of PPIs has been recognized as a major global concern, contributing to excess health care expenditure and polypharmacy.^5^ Furthermore, PPIs have been associated with an increased risk of various diseases, such as pneumonia, Clostridium difficile infection, hypomagnesemia, and interstitial nephritis.^6^, ^7^, ^8^, ^9^

PPI use is also associated with adverse kidney outcomes, including acute kidney injury, incident CKD, and progression to kidney failure.^10^^,^^11^ A *post hoc* analysis of a large randomized clinical trial reported that pantoprazole use was associated with a faster eGFR decline compared with placebo.^12^ Accordingly, the Kidney Disease: Improving Global Outcomes 2024 guideline for CKD lists PPIs as medications that warrant careful evaluation for potential nephrotoxicity.^13^ In clinical practice, however, PPIs are often continued for prolonged periods despite uncertain clinical benefit. It remains unknown whether appropriate early discontinuation of PPIs could reduce the risk of kidney outcomes.

In this target trial emulation study, we first aimed to replicate previously reported association between PPI use and the risk of kidney failure using an active comparator new user design. We then estimated the causal effect of early PPI discontinuation on kidney outcomes using a clone-censor-weighting approach.

## Methods

### Study Design and Target Trial Emulation

We implemented the target trial emulation framework, which explicitly specifies the design components of a hypothetical trial and minimizes avoidable biases in observational studies to improve causal inference and increase confidence in the validity of real-world evidence.^14^^,^^15^ Specifically, we emulated the following 2 target randomized controlled trials to assess the effect of PPI use on kidney outcome: (i) a head-to-head trial comparing new use of PPIs versus histamine_2_ receptor antagonists (H_2_ blockers), analyzed using an active comparator new user design^16^; and (ii) a trial comparing strategies of PPI continuation versus discontinuation, estimated using the clone-censor-weighting approach.^17^^,^^18^ Both emulations shared the common data source and outcome, but differed in treatment strategies and statistical approach.

This study adhered to the principles outlined in the Declaration of Helsinki, and the protocol was approved by the ethics committee of each participating hospital (approval number: 21513). The need for informed consent was waived because of the retrospective nature of the study.

### Data Collection and Study population

All data used in the present analyses were obtained from the Osaka Consortium for Kidney Disease Research database, a retrospective multicenter cohort study, that included patients who attended the outpatient departments of nephrology at 5 hospitals in Osaka, Japan (Ikeda City Hospital, Osaka General Medical Center, Osaka University Hospital, Sakai City Medical Center, and Toyonaka Municipal Hospital), from January 2005 to March 2022. Demographic and clinical characteristics, laboratory measurements, and prescription data from both outpatient and inpatient settings at each hospital were automatically extracted using an electronic data capture system integrated with electronic medical records and combined into a single dataset for analysis. Blood pressure measurements and histories of cardiovascular disease—coronary artery diseases, heart failure, atrial fibrillation, and stroke—were manually collected through electronic medical record review. Laboratory and prescription data were organized on a monthly basis. The detailed methods of the study and data collection have been described elsewhere.^19^^,^^20^

The present analysis included patients who met the following criteria: (ⅰ) aged ≥ 20 years; (ⅱ) an eGFR at baseline of 10 to 60 ml/min per 1.73 m^2^; (ⅲ) followed up in the departments for ≥ 30 days; (ⅳ) no history of kidney replacement therapy; and (ⅴ) received prescriptions for either PPIs or H_2_ blockers for ≥ 30 consecutive days during the observation period. Prevalent users, defined as those already prescribed either PPIs or H_2_ blockers at the time of cohort registration, were excluded to restrict the analysis to new users.

### Treatment Assignment

In the active comparator new user design, each patient was assigned to the treatment group based on the first prescription record of either PPIs or H_2_ blockers received during the observation period.

In the clone-censor-weighting approach, new users of PPIs were classified into 2 treatment strategies according to their subsequent use. A grace period of 90 days after initiation was allowed to define treatment continuation. Patients who discontinued PPI therapy within this 90-day grace period were assigned to the discontinuation group, whereas those who continued therapy beyond 90 days were assigned to the continuation group. Discontinuation was defined as having no prescriptions for a period of ≥ 30 days following the previous supply.

### Study Outcome and Follow-up

The outcome of interest was KFRT, defined as the initiation of maintenance hemodialysis, peritoneal dialysis, or kidney transplantation. The date of KFRT was identified from patient registries at each participating institution, which recorded demographic and clinical information of patients who progressed to KFRT. Additionally, all-cause mortality was assessed, and the dates of death were obtained from the electronic data capture system.

Patients were followed from the index date, when PPIs or H_2_blockers were first prescribed, until the progression to KFRT, death, loss to follow-up, 5 years after the index date, or the end of the study period (March 31, 2022), whichever occurred first. Loss to follow-up was defined as cessation of attendance at the participating institutions, and patients lost to follow-up were censored at the time of their last visit.

### Covariates

Baseline covariates were assessed using the most recent measurements or records available within the 30 days before the first prescription of either PPIs or H_2_ blockers. Baseline covariates included age, sex, body mass index, systolic blood pressure, diabetes mellitus, cardiovascular disease, laboratory measurements (hemoglobin, sodium, potassium, corrected calcium, phosphate, magnesium, serum urate, albumin, total cholesterol, eGFR, log-transformed C-reactive protein, and log-transformed urinary protein-creatinine ratio), and prescriptions (angiotensin-converting enzyme inhibitors or angiotensin II receptor blockers, calcium channel blockers, beta-blockers, loop diuretics, thiazide diuretics, and mineralocorticoid receptor antagonists, nonsteroidal anti-inflammatory drugs, steroids, antithrombotics [including both antiplatelets and anticoagulants]). The laboratory measurements and prescriptions were also treated as time-varying covariates. The annual number of serum creatinine measurements and the number of hospitalizations in the preceding year were also included as indicators of monitoring intensity and overall health care utilization.

Diabetes mellitus was defined as baseline HbA1c ≥ 6.5% or use of glucose-lowering drugs at baseline. Serum calcium was corrected for serum albumin levels as follows: corrected calcium (mg/dl) = total calcium + 0.8 × (4.0 − albumin), if albumin <4.0 (g/dl).^21^ The eGFRs were calculated using the following Japanese standard formula: 194 × Scr^−1.094^ × age^−0.287^ (if female, × 0.739).^22^

### Statistical Analysis

Baseline characteristics are presented as frequencies (percentages) for categorical variables, and as mean (SD) or median (interquartile range [IQR]) for continuous variables depending on the distributions.

#### Active Comparator New User Design

We examined the association of the initiation of PPIs versus H_2_ blockers with study outcomes using an active comparator new user design. H_2_ blockers were selected as the active comparator because they share similar therapeutic indications with PPIs, enabling a clinically relevant comparison. To address potential confounding by indication, we estimated the probability of initiating PPIs versus H_2_ blockers using logistic regression models including all baseline covariates described above. Each patient in the PPI group was weighted by 1/propensity score, and each patient in the H_2_ blocker group by 1/(1-propensity score). Weights were stabilized using the marginal probability of the received treatment as the numerator of the weights. Outcome analyses were conducted using inverse probability of treatment weighting-Cox proportional hazards models. To account for the competing risk of death, a Fine-Gray subdistribution hazard model was also applied as a complementary analysis. Details of the target trial protocol of comparing PPIs versus H_2_ blockers are summarized in Supplementary Table S1.

#### Clone-Censor-Weighting

When examining the association of PPI discontinuation versus continuation with outcomes in observational data, indication bias and immortal time bias are major concerns. For example, in a conventional time-to-event analysis, individuals who experienced outcome events before discontinuing PPIs would be excluded from the discontinuation group in the analysis. This creates a period of guaranteed event-free-survival until PPI discontinuation only in the discontinuation group. To address these issues, we adopted the clone-censor-weighting approach to emulate the target trial comparing strategies of PPI discontinuation versus continuation. The clone-censor-weighting conceptually creates and analyzes a balanced pseudo-population, in which confounding by indication and immortal time bias is minimized.^23^ The present pseudo-population was constructed as follows (Supplementary Figure S1). First, each individual was cloned into 2 copies. One copy was assigned to the strategy of discontinuing PPIs within a 90-day grace period after treatment initiation (PPI discontinuation arm), and the other copy was assigned to the strategy of continuing PPIs beyond the grace period (PPI continuation arm). The cloning approach provides the identical backgrounds in both arms, and enables to analyze all outcome events within the grace period (Supplementary Figure S1). Second, individuals were artificially censored when they deviated from their assigned strategy—those in the discontinuation arm were censored at the end of the 90-day grace period if they had not discontinued PPIs during the period, whereas those in the continuation arm were censored at the time of PPI discontinuation if discontinuation occurred within the grace period. Those who discontinued PPIs “after” the grace period were not censored, and were treated as if they had continued them until the end of follow-up to provide a conservative estimate of the causal effect (intention-to-treat-like analysis). Because such artificial censoring is informative and may introduce selection bias, we applied time-varying inverse probability of censoring weighting (IPCW). The IPCWs were used to create a balanced pseudo-population (IPCW-weighted population), in which no individuals deviated from their assigned treatment strategy nor thus censored artificially. At each time point throughout the grace period, the IPCW was estimated as the inverse of the probability of remaining uncensored artificially, using a logistic regression model including all baseline and time-varying covariates, as appropriate. IPCWs were stabilized by multiplying them by the probability of remaining uncensored artificially estimated by a separate logistic regression model including baseline covariates only.

HRs were estimated using IPCW-weighted pooled logistic regression models, which emulate time-dependent Cox regression models while allowing incorporation of time-varying weights.^24^ Follow-up time was included as linear and quadratic terms. The 95% CIs were based on 2.5th and 97.5th percentiles of the empirical distribution of the point estimates, obtained from nonparametric bootstrap resampling with 500 iterations. Details of the target trial protocol of comparing PPI discontinuation versus continuation are summarized in Supplementary Table S2.

#### Imputation for Missing Data

Missing values in baseline covariates were imputed using multiple imputation by chained equations.^25^ Given that the covariates with missing data (body mass index, systolic blood pressure, hemoglobin, sodium, potassium, corrected calcium, phosphate, magnesium, urate, albumin, total cholesterol, eGFR, C-reactive protein, urinary protein-creatinine ratio) were all continuous, linear regression imputation was applied. Five imputed datasets were generated, analyzed separately, and then combined using Rubin’s rules. Missing data during follow-up were imputed by the last observation carried forward method.

#### Subgroup and Sensitivity Analyses

We performed subgroup analysis to assess effect modification by baseline covariates, including age (≥ 75 vs. ≤ 74 years), sex, diabetes mellitus, cardiovascular disease, hemoglobin (≥ 11.0 vs. < 11.0 g/dl), magnesium (≥ 2.0 vs. < 2.0 mg/dl), eGFR (≥ 30 vs. < 30 ml/min per 1.73 m^2^), urinary protein-creatinine ratio (≥ 1.0 vs. < 1.0 g/gCre), and medications(steroids, antithrombotics).

Several sensitivity analyses were performed to assess the robustness of the primary findings. First, we evaluated the impact of varying the grace period for PPI discontinuation from the primary definition of 90 days. Second, we performed a separate clone–censor–weighting analysis in which individuals were additionally censored at the time of PPI discontinuation even after the grace period (On-treatment-like analysis). Finally, we repeated the clone-censor-weighting analysis using H_2_ blockers as a negative control exposure to assess potential residual confounding and nonspecific treatment effects.

Statistical tests were 2-sided and statistical significance was defined as *P* < 0.05. All statistical analyses were performed using StataNow/Multiprocessor (version19.5; Stata Corp, College Station, TX).

## Results

### Study Population

In the active comparator new user analysis, 3512 patients initiated PPIs first, whereas 1102 initiated H_2_ blockers first (Supplementary Figure S2). At treatment initiation (baseline), the mean (SD) age and eGFR were 71 (13) and 30 (17) ml/min per 1.73 m^2^, respectively. Baseline characteristics before and after weighting are summarized in Supplementary Table S3. PPI users were older, had lower eGFR, and a higher prevalence of the use of medications, including antihypertensive agents, diuretics, steroids, and antithrombotics. After weighting, baseline characteristics were all well-balanced between the 2 groups (Supplementary Figure S3).

Of 4187 patients who initiated PPIs during follow-up and were eligible for the clone-censor-weighting analysis, 1254 discontinued PPIs within 90 days, whereas 2933 continued PPIs beyond 90 days (Supplementary Figure S4). Patient characteristics at PPI initiation are shown in Table 1. Demographic and laboratory data were generally comparable between the 2 strategy groups. Concomitant medication use was generally more frequent in the PPI continuation group.

**Table 1:** Baseline characteristics of new users of PPIs at treatment initiation

Characteristics	Missing data	Overall	Discontinuation	Continuation
	*n* (%)	*N* = 4187	*n* = 1254	*n* = 2933
Demographic characteristics				
Men, *n* (%)	0 (0)	2564 (61)	794 (63)	1770 (60)
Age, yrs	0 (0)	71 (13)	73 (12)	71 (13)
Body mass index, kg/m^2^	205 (5)	22.3 (4.4)	22.4 (4.4)	22.2 (4.3)
Systolic blood pressure, mm Hg	445 (11)	136 (23)	138 (24)	136 (23)
Diabetes mellitus, *n* (%)	0 (0)	1724 (41)	513 (41)	1211 (41)
Previous cardiovascular disease, *n* (%)	0 (0)	1383 (33)	463 (37)	920 (31)
Number of hospitalizations in the preceding yr, *n*	0 (0)	0.4 (0.8)	0.4 (0.8)	0.4 (0.7)
Serum creatinine measurements per yr, *n*/yr, median (IQR)	0 (0)	12.8 (7.9–21.0)	11.4 (6.8–17.8)	13.4 (8.5–22.2)
Laboratory data				
Hemoglobin, g/dl	22 (1)	11.2 (2.2)	11.2 (2.3)	11.1 (2.2)
Sodium, mEq/l	24 (1)	139 (4)	139 (4)	139 (4)
Potassium, mEq/l	22 (1)	4.4 (0.6)	4.4 (0.6)	4.4 (0.6)
Corrected calcium, mg/dl^a^	239 (6)	9.3 (0.8)	9.3 (0.7)	9.3 (0.9)
Phosphate, mg/dl	648 (15)	3.7 (1.0)	3.7 (0.9)	3.7 (1.0)
Magnesium, mg/dl	1783 (43)	2.1 (0.4)	2.1 (0.4)	2.1 (0.4)
Albumin, g/dl	54 (1)	3.5 (0.7)	3.5 (0.7)	3.4 (0.7)
eGFR, ml/min per 1.73 m^2^	0 (0)	30 (17)	30 (17)	30 (17)
Total cholesterol, mg/dl	901 (22)	191 (63)	187 (59)	192 (65)
Serum urate, mg/dl	252 (6)	6.7 (2.0)	6.6 (1.9)	6.7 (2.0)
C-reactive protein, mg/dl, median (IQR)	149 (4)	0.2 (0.1–1.0)	0.2 (0.1–1.0)	0.2 (0.1–1.0)
UPCR, g/gCre, median (IQR)	634 (15)	0.9 (0.2–3.1)	0.8 (0.2–2.8)	0.9 (0.2–3.2)
Prescriptions				
ACEIs/ARBs, *n* (%)	0 (0)	1709 (41)	464 (37)	1245 (42)
Calcium channel blockers, *n* (%)	0 (0)	2180 (52)	583 (46)	1597 (54)
Beta blockers, *n* (%)	0 (0)	1126 (27)	321 (26)	805 (27)
Loop diuretics, *n* (%)	0 (0)	1640 (39)	409 (33)	1231 (42)
Thiazide diuretics, *n* (%)	0 (0)	450 (11)	112 (9)	338 (12)
MRAs, *n* (%)	0 (0)	508 (12)	126 (10)	382 (13)
Systemic steroids, *n* (%)	0 (0)	1039 (25)	217 (17)	822 (28)
NSAIDs, *n* (%)	0 (0)	116 (3)	39 (3)	77 (3)
Antithrombotics, *n* (%)	0 (0)	1207 (29)	368 (29)	839 (29)

ACEI, angiotensin-converting enzyme inhibitor; ARB, angiotensin II receptor blocker; eGFR, estimated glomerular filtration rate; IQR, interquartile range; MRA, mineralocorticoid receptor antagonist; NSAID, nonsteroidal anti-inflammatory drugs; PPI, proton pump inhibitor; UPCR, urinary protein-creatinine ratio.

Data are presented as the mean (SD) unless otherwise indicated.

SI conversion factors: To convert hemoglobin, albumin, and C-reactive protein to g/l, multiply by 10; sodium and potassium to mmol/l, multiply by 1.0; and calcium to mmol/l, multiply by 0.25; phosphate to mmol/l, multiply by 0.323; magnesium to mmol/l, multiply by 0.4114; total cholesterol to mmol/l, multiply by 0.0259; urate to mmol/l, multiply by 0.0595.

a Serum calcium was corrected for serum albumin levels using the formula: corrected calcium (mg/dl) = total calcium + 0.8 × (4.0 − albumin), if albumin < 4.0 (g/dl).

### Comparison of Clinical Outcomes Between New Users of PPIs and H_2_ Blockers

During a median (IQR) follow-up of 2.1 (0.7–4.6) years, 509 patients progressed to KFRT and 738 died among new users of PPIs, whereas 145 progressed to KFRT and 224 died among new users of H_2_ blockers. The weighted incidence rate of KFRT was higher in the PPI new users (49.7 [95% CI: 45.4–54.4] per 1000 person-years) than in the H_2_ blocker new users (33.8 [95% CI: 28.0–41.0] per 1000 person-years) (Table 2 and Supplementary Figure S5A). PPI initiation was significantly associated with an increased risk of KFRT compared with H_2_ blocker initiation (weighted HR: 1.39; 95% CI: 1.13–1.72; Table 2). A competing risk analysis using the Fine-Gray subdistribution hazard model yielded consistent results (Supplementary Table S4).

**Table 2:** Associations of PPIs versus H_2_ blockers with KFRT and all-cause mortality

Outcomes	Treatment strategies	No. of events	Weighted incidence rate (95% CI)^a^	Unweighted HR (95% CI)	Weighted HR (95% CI)^b^
KFRT	H_2_ blockers	145	33.8 (28.0–41.0)	Reference	Reference
	PPIs	509	49.7 (45.4–54.4)	1.57 (1.31–1.89)	1.39 (1.13–1.72)
All-cause mortality	H_2_ blockers	224	59.7 (51.5–69.5)	Reference	Reference
	PPIs	738	69.4 (64.3–74.9)	1.46 (1.25–1.69)	1.10 (0.93–1.30)

CI, confidence interval; HR, hazard ratio; H_2_ blocker, histamine_2_ receptor antagonist; KFRT, kidney failure with replacement therapy; PPI, proton pump inhibitor.

a Incidence rates were expressed as the number of events per 1000 person-years.

b Weighted hazard ratios were estimated using the inverse probability of treatment weights derived from the following covariates: sex, age, body mass index, systolic blood pressure, history of diabetes mellitus, history of cardiovascular disease, the number of hospitalizations in the preceding year, the annual number of serum creatinine measurements, hemoglobin, sodium, potassium, corrected calcium, phosphate, magnesium, serum urate, albumin, total cholesterol, estimated glomerular filtration rate, log-transformed C-reactive protein, log-transformed urinary protein-creatinine ratio, prescriptions of angiotensin-converting enzyme inhibitors/angiotensin II receptor blockers, calcium channel blockers, beta-blockers, loop diuretics, thiazide diuretics, and mineralocorticoid receptor antagonists, nonsteroidal anti-inflammatory drugs, steroids, antithrombotics.

PPI initiation was consistently associated with higher hazards of KFRT across prespecified subgroups, although potential effect modification by magnesium levels was observed. The association was more pronounced in patients with lower baseline magnesium levels (Supplementary Figure S6).

The risk of all-cause mortality was not significantly different between PPI initiation and H_2_ blocker initiation (weighted HR: 1.10; 95% CI: 0.93–1.30; Supplementary Figure S5B and Table 2).

### Causal Effects of PPI Discontinuation on Clinical Outcomes

Over a median (IQR) follow-up of 2.1 (0.7–4.6) years, 564 replicates (176 in the discontinuation group and 388 in the continuation group) initiated KFRT, and 881 (358 in the discontinuation group and 523 in the continuation group) died. The distribution of the IPCW weights is summarized in Supplementary Table S5.

**Figure 1:**
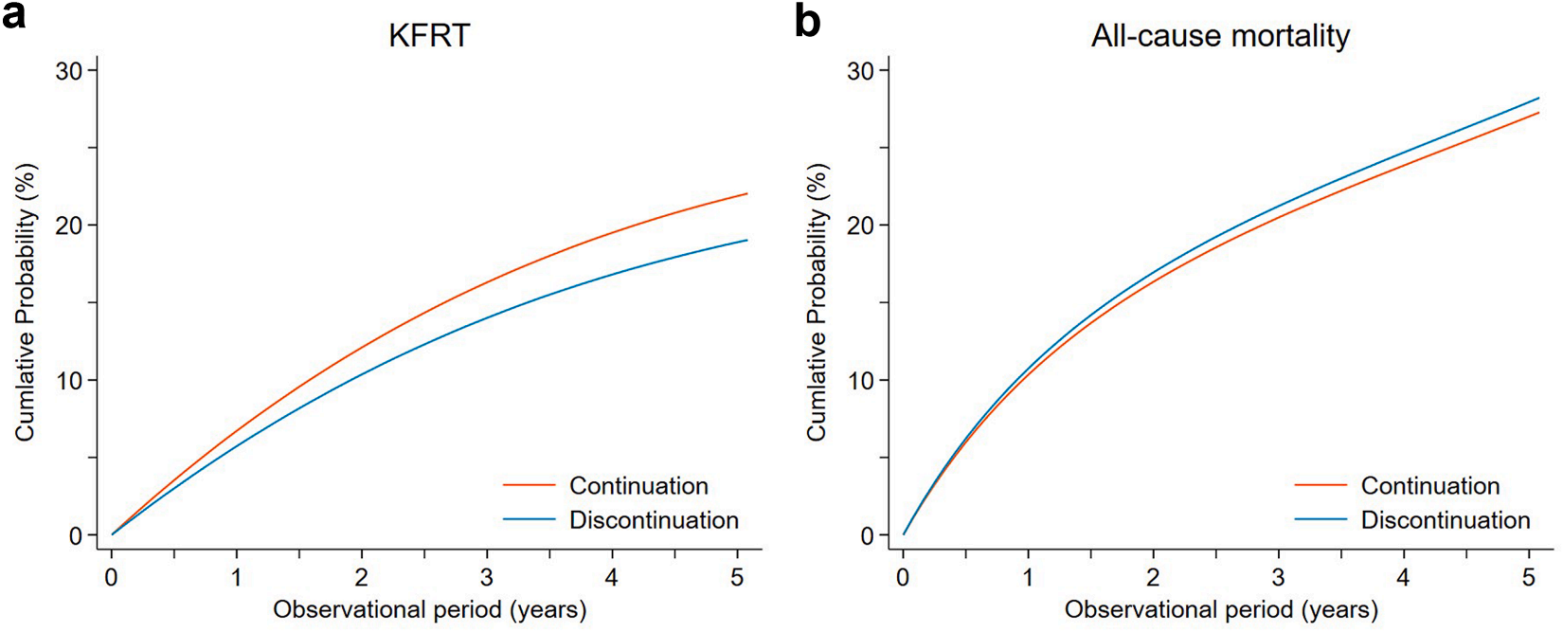
Weighted cumulative probability curves of KFRT and all-cause mortality according to PPI treatment strategy. The median length of follow-up was 1.9 years (interquartile range: 0.6–4.4 years). The PPI discontinuation group showed a significantly lower cumulative probability of KFRT than the continuation group (a). The cumulative probability of all-cause mortality was comparable between the groups (b). KFRT, kidney failure with replacement therapy; PPI, proton pump inhibitor.

The estimated 5-year absolute risk of KFRT was 18.8% (95% CI: 16.4–21.2) in the discontinuation group and 21.9% (95% CI: 20.3–23.6) in the continuation group, corresponding to an absolute risk difference of -3.0% (95% CI: −5.7 to −0.6) (Figure 1a and Table 3). The hazard of KFRT was 15% lower with the discontinuation strategy compared with the continuation strategy (HR: 0.85; 95% CI: 0.72–0.97).

**Table 3:** Associations of PPI discontinuation versus continuation with KFRT and all-cause mortality

Outcomes	Treatment strategies	No. of events n the emulated trial	5-year absolute risk (%) (95% CI)	5-year risk difference (%) (95% CI)	Hazard ratio (95% CI)
KFRT	Continuation	388	21.9 (20.3–23.6)	Reference	Reference
	Discontinuation	176	18.8 (16.4–21.2)	-3.0 (-5.7 to -0.6)	0.85 (0.72–0.97)
All-cause mortality	Continuation	523	27.3 (25.5–29.1)	Reference	Reference
	Discontinuation	358	28.0 (25.1–30.8)	0.7 (-1.9 to 3.4)	1.03 (0.92–1.15)

CI, confidence interval; HR, hazard ratio; KFRT, kidney failure with replacement therapy; PPI, proton pump inhibitor.

Analyses were adjusted through the inverse probability of censoring weights derived from the following covariates: sex, age, body mass index, systolic blood pressure, history of diabetes mellitus, history of cardiovascular disease, the number of hospitalizations in the preceding year, the annual number of serum creatinine measurements, hemoglobin, sodium, potassium, corrected calcium, phosphate, magnesium, serum urate, albumin, total cholesterol, estimated glomerular filtration rate, log-transformed C-reactive protein, log-transformed urinary protein-creatinine ratio, prescriptions of angiotensin-converting enzyme inhibitors/angiotensin II receptor blockers, calcium channel blockers, beta-blockers, loop diuretics, thiazide diuretics, and mineralocorticoid receptor antagonists, nonsteroidal anti-inflammatory drugs, steroids, and antithrombotics.

**Figure 2:**
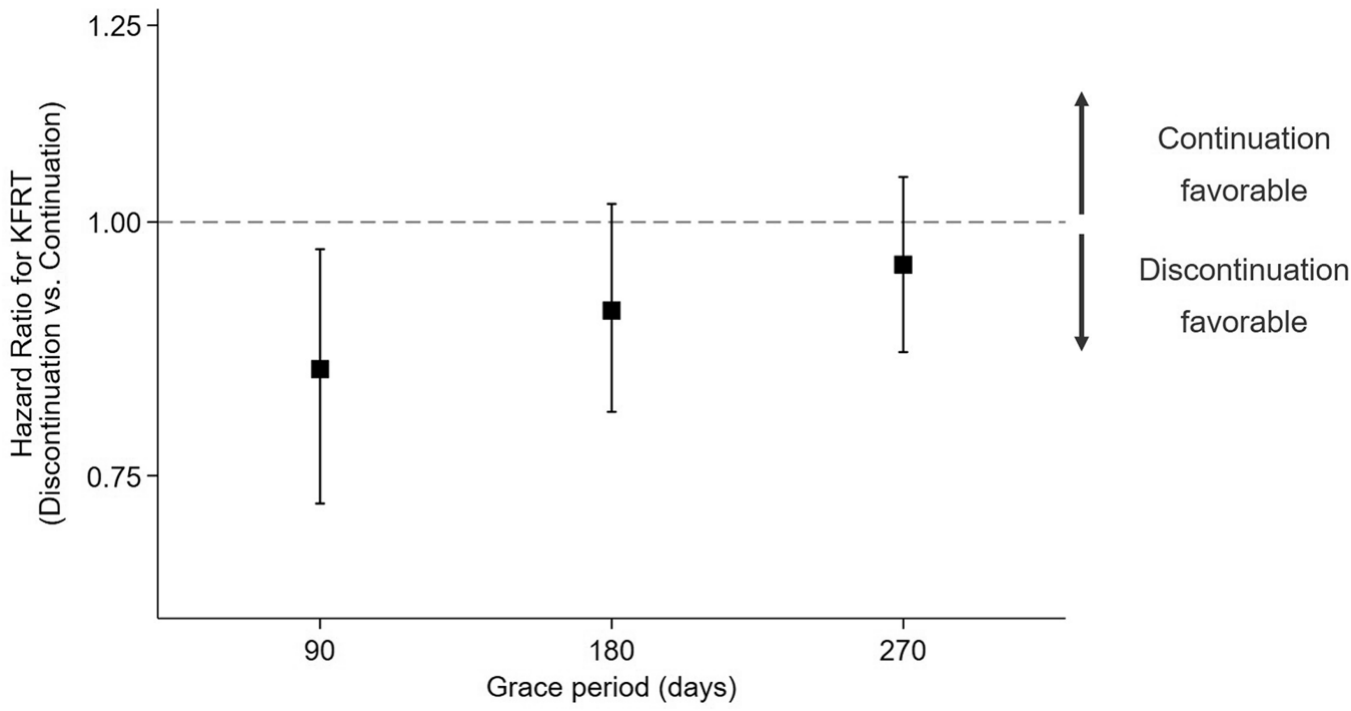
Associations of PPI discontinuation versus continuation with KFRT according to the grace period for PPI discontinuation. The dots represent hazard ratios for kidney failure with replacement therapy in PPI discontinuation versus continuation. The error bars represent the 95% confidence intervals. Hazard ratios were estimated using the inverse probability of censoring weights derived from the following covariates: sex, age, body mass index, systolic blood pressure, history of diabetes mellitus, history of cardiovascular disease, the number of hospitalizations in the preceding year, the annual number of serum creatinine measurements, hemoglobin, sodium, potassium, corrected calcium, phosphate, magnesium, serum urate, albumin, total cholesterol, estimated glomerular filtration rate, log-transformed C-reactive protein, log-transformed urinary protein-creatinine ratio, prescriptions of angiotensin-converting enzyme inhibitors/angiotensin II receptor blockers, calcium channel blockers, beta-blockers, loop diuretics, thiazide diuretics, and mineralocorticoid receptor antagonists, nonsteroidal anti-inflammatory drugs, steroids, antithrombotics. HR, hazard ratio; KFRT, kidney failure with replacement therapy; PPI, proton pump inhibitor.

When the grace period was extended beyond the original 90-day definition to accommodate later PPI discontinuation, the HR for KFRT was progressively attenuated toward the null and lost statistical significance at 180 days or longer (Figure 2).

**Figure 3:**
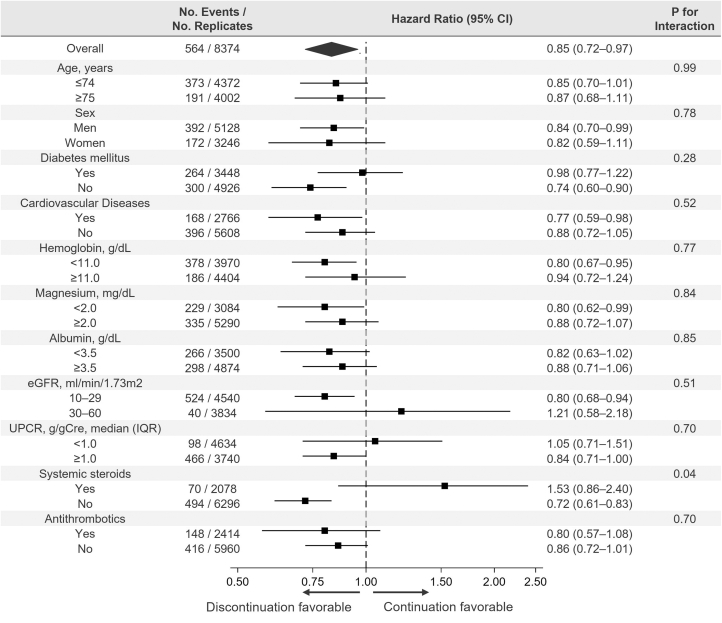
Associations of PPI discontinuation versus continuation with KFRT across different subgroups of patients. The dots represent hazard ratios for kidney failure with replacement therapy in PPI discontinuation versus continuation. The error bars represent the 95% confidence intervals. Hazard ratios were estimated using the inverse probability of censoring weights derived from the following covariates: sex, age, body mass index, systolic blood pressure, history of diabetes mellitus, history of cardiovascular disease, the number of hospitalizations in the preceding year, the annual number of serum creatinine measurements, hemoglobin, sodium, potassium, corrected calcium, phosphate, magnesium, serum urate, albumin, total cholesterol, estimated glomerular filtration rate, log-transformed C-reactive protein, log-transformed urinary protein-creatinine ratio, prescriptions of angiotensin-converting enzyme inhibitors/angiotensin II receptor blockers, calcium channel blockers, beta-blockers, loop diuretics, thiazide diuretics, and mineralocorticoid receptor antagonists, nonsteroidal anti-inflammatory drugs, steroids, antithrombotics. eGFR, estimated glomerular filtration rate; KFRT, kidney failure with replacement therapy; PPI, proton pump inhibitor; UPCR, urinary protein-creatinine ratio.

In prespecified subgroup analyses, the discontinuation group consistently showed a lower hazard of KFRT across all subgroups except for baseline steroid use. The lower hazard was observed in patients not receiving steroids (HR: 0.72; 95% CI: 0.61–0.83), but not in those receiving steroids (HR: 1.53; 95% CI: 0.86–2.40; *P* for interaction = 0.04), although the *P*-value for interaction was not adjusted for multiple comparisons (Figure 3). On-treatment analyses, in which patients were censored at the time of deviation from the assigned strategy after the grace period, yielded consistent results (Supplementary Table S6).

In contrast, the estimated 5-year absolute risk of all-cause mortality was comparable between the discontinuation and continuation groups (absolute risk difference: 0.7%; 95% CI: −1.9 to 3.4) (Figure 1b and Table 3). Similarly, there was no significant difference in the hazard of all-cause mortality between the 2 groups (HR: 1.03; 95% CI: 0.92–1.15).

In an analysis using H_2_ blockers as the exposure, neither the hazard of KFRT (HR: 1.02; 95% CI: 0.76–1.30) nor that of all-cause mortality (HR: 0.92; 95% CI: 0.75–1.11) differed significantly between the discontinuation and continuation groups (Supplementary Table S7).

## Discussion

In this large multicenter cohort of patients with CKD, we estimated the causal effects of PPI initiation and subsequent discontinuation on clinical outcomes using a target trial emulation framework. Consistent with previous evidence, PPI initiation was associated with a significantly higher risk of KFRT than H_2_ blocker initiation. Notably, among PPI users, discontinuation of PPIs within 90 days after initiation showed a 15% lower hazard of KFRT compared with that of continuation. This potential benefit on the kidney outcome was attenuated as the period until PPI discontinuation was extended. PPI discontinuation was not significantly associated with all-cause mortality.

Previous studies, including meta-analyses of observational studies and a *post hoc* analysis of a randomized controlled trial, have reported associations between PPI use and increased risk of adverse kidney outcomes.^10^, ^11^, ^12^ Xie *et al.*^26^^,^^27^ showed an association between the cumulative duration of PPI use and adverse kidney outcomes. However, because follow-up was initiated at the time of PPI discontinuation, their findings do not directly inform clinical decision-making for patients who received ongoing PPI therapy. In contrast, we initiated follow-up at the time of PPI initiation using a target trial emulation framework with a clone-censor weighting approach to address various biases inherent to observational data, such as time-varying confounding, immortal time bias, and selection bias. In this analytical model, we showed that early PPI discontinuation was associated with a lower risk of KFRT. Although interventional studies are warranted to validate the causality, these findings may have implications for optimizing PPI use in patients with CKD.

Several mechanisms underlying the potential nephrotoxicity of PPIs have been proposed. First, PPIs have been implicated in acute interstitial nephritis, an idiosyncratic cell-mediated immune response.^9^ Since drug-induced tubulointerstitial nephritis frequently presents with nonspecific or subtle clinical manifestations, it is often overlooked in routine clinical practice.^28^ However, persistent tubulointerstitial injury can lead to irreversible interstitial fibrosis and CKD progression. Second, chronic PPI use induces hypomagnesemia because of impaired intestinal magnesium absorption,^29^^,^^30^ which might contribute to tubular injury and fibrosis.^31^ Indeed, lower serum magnesium levels are associated with an increased risk of adverse kidney outcomes in patients with CKD.^32^^,^^33^ Notably, the increased risk of KFRT after PPI initiation, compared with H_2_ blocker initiation, was more pronounced in a subgroup of patients with lower serum magnesium levels, suggesting the possibility that PPIs further exacerbate hypomagnesemia to levels that contribute to CKD progression. Therefore, caution is required when PPIs are administered for patients with low serum magnesium levels.

Pharmacological therapies for CKD have evolved dramatically over the past decade. Besides angiotensin-converting enzyme inhibitors and angiotensin receptor blockers, sodium-glucose co-transporter 2 inhibitors became a cornerstone in the prevention of CKD progression.^34^^,^^35^ Nonsteroidal mineralocorticoid receptor antagonists and glucagon-like peptide-1 receptor agonists have also demonstrated kidney-protective effects in diabetic kidney disease.^36^, ^37^, ^38^ Accordingly, their initiation and optimization are widely recommended in the current clinical practice. In contrast, less attention is paid to reassessment of ongoing therapies even when they include potentially nephrotoxic drugs. Discontinuing medications may be more challenging than initiating them because of clinical inertia where prescribing decisions tend to favor addition over subtraction.^39^^,^^40^ This clinical inertia also contributes to the burden of polypharmacy among patients with CKD.^41^ Our findings underscore the importance of reassessment and deprescribing of PPIs without clear indications as part of kidney-protective strategies in patients with CKD, in addition to reducing polypharmacy.

The favorable association between early PPI discontinuation and kidney outcomes was not observed among steroid users. This heterogeneity may have arisen from differences in the underlying kidney diseases between steroid users and nonusers. Since patients requiring steroid therapy are likely to have more active or severe disease, the potential benefit of PPI discontinuation may be obscured in this subgroup. Thus, clinical decisions regarding PPI discontinuation in steroid users may need to be individualized based on the clinical context.

Our study has some limitations. First, causality cannot be established because of the observational design of this study, even though we applied a target trial emulation framework with an active comparator new user design and a clone-censor weighting approach. Second, residual confounding cannot be ruled out despite extensive covariate adjustment. For example, we acknowledge that potential poorer underlying health or socioeconomic status among PPI users compared with H_2_ blocker users could, at least partly, explain the substantially higher risk of KFRT observed among PPI users. However, such confounding is likely to be modest, if present, given the absence of a statistically significant between-group difference in the risk of all-cause mortality (weighted HR in PPI users vs. H_2_ blocker users: 1.10; 95% CI: 0.93–1.30). In other words, the observed association with KFRT is unlikely to be merely a reflection of differences in underlying health or socioeconomic status between the 2 groups. Furthermore, H_2_ blockers, a negative control exposure, was not associated with KFRT (Supplementary Table S7), implying that the observed association with PPI discontinuation is unlikely to reflect nonspecific effects of treatment discontinuation. Third, since PPI discontinuation and continuation were identified from prescription data, some degree of exposure misclassification is inevitable. As such misclassification generally biases the estimates toward the null, our findings would not mislead to an overestimation. Fourth, the specific indications for initiating PPIs and the reasons for their discontinuation were unavailable. Fifth, sodium-glucose cotransporter 2 inhibitor, approved for the treatment of CKD in Japan in 2021, was not incorporated into the present analyses because of the study period (from January 2005 to March 2022). Finally, the generalizability of our findings warrants careful consideration. The study analyzed outpatients with CKD receiving nephrology care, who may represent patients with more advanced CKD and may receive more intensive kidney-protective management than those with earlier-stage CKD managed in primary care settings. In addition, because the cohort exclusively included Japanese patients, the present findings cannot necessarily be extrapolated to other ethnic populations, given potential differences in genetic background, dietary habits, and drug metabolism.

In conclusion, using a target trial emulation in a large multicenter CKD cohort, we found that PPI initiation was associated with a higher risk of KFRT compared with H_2_ blocker initiation. Furthermore, among patients who initiated PPIs, early discontinuation of PPIs exhibited a lower risk of KFRT than continued use. These findings underscore the importance of careful reassessment and deprescribing of PPIs without clear clinical indications, suggesting that appropriate discontinuation of PPIs may contribute to a valuable component of kidney protection strategies.

## Disclosure

All the authors declared no competing interests.

## Data Availability Statement

The data underlying this article are not publicly available because of the privacy of the study participants. Deidentified individual participant data, analytical datasets, and STATA programs used in this study are available upon reasonable request to the corresponding author.

## Author Contributions

YK and TO contributed equally to this study. YK and YS conceptualized the study. YK analyzed the data. YK and YS drafted the manuscript. HK, RU, SF, TO, YD, RY, IM, MM, J-YK, and YI reviewed and revised the manuscript. NH, YK, SY, and MY contributed to the data acquisition. All the authors have read and approved the final version of this manuscript.

## Supplementary Material

Supplementary File (PDF) **Figure S1.** Process of the cloning, censoring, and weighting method. **Figure S2.** Flow diagram of the study patients for the emulated trial comparing PPIs and H_2_ blockers. **Figure S3.** Balance of baseline characteristics with standardized mean difference before and after weighting. **Figure S4.** Flow diagram of the study patients for the emulated trial comparing PPI discontinuation and continuation. **Figure S5.** Weighted cumulative incidence curves of KFRT and all-cause mortality for PPI versus H_2_ blocker. **Figure S6.** Associations of PPIs versus H_2_ blockers with KFRT across different subgroups of patients. **Table S1.** Brief protocol of the target trial and its emulation using the Osaka Consortium for Kidney Disease Research database: Comparison between PPI and H_2_ blocker initiation. **Table S2.** Brief protocol of the target trial and its emulation using the Osaka Consortium for Kidney Disease Research database: Comparison between PPI discontinuation and continuation. **Table S3.** Baseline characteristics of new users of PPIs and H_2_ blockers at treatment initiation before and after IPTW. **Table S4.** Associations of PPIs versus H2 blockers with KFRT using a Fine-Grey subdistribution hazard model accounting for the competing risk of death. **Table S5.** Distribution of the inverse probability of censoring weights in the clone-censor-weighting analysis. **Table S6.** Associations of H2 blocker discontinuation versus continuation with KFRT and all-cause mortality in on-treatment analyses. **Table S7.** Associations of H_2_ blocker discontinuation versus continuation with KFRT and all-cause mortality. Statistical program code for the clone-censor-weighting analysis. STROBE Checklist.

## Footnotes

Supplementary File (PDF)
**Figure S1.** Process of the cloning, censoring, and weighting method. **Figure S2.** Flow diagram of the study patients for the emulated trial comparing PPIs and H_2_ blockers **Figure S3.** Balance of baseline characteristics with standardized mean difference before and after weighting **Figure S4.** Flow diagram of the study patients for the emulated trial comparing PPI discontinuation and continuation **Figure S5.** Weighted cumulative incidence curves of KFRT and all-cause mortality for PPI versus H_2_ blocker **Figure S6.** Associations of PPIs versus H_2_ blockers with KFRT across different subgroups of patients **Table S1.** Brief protocol of the target trial and its emulation using the Osaka Consortium for Kidney Disease Research database: Comparison between PPI and H_2_ blocker initiation. **Table S2.** Brief protocol of the target trial and its emulation using the Osaka Consortium for Kidney Disease Research database: Comparison between PPI discontinuation and continuation. **Table S3.** Baseline characteristics of new users of PPIs and H_2_ blockers at treatment initiation before and after IPTW. **Table S4.** Associations of PPIs versus H2 blockers with KFRT using a Fine-Grey subdistribution hazard model accounting for the competing risk of death. **Table S5.** Distribution of the inverse probability of censoring weights in the clone-censor-weighting analysis. **Table S6.** Associations of H2 blocker discontinuation versus continuation with KFRT and all-cause mortality in on-treatment analyses. **Table S7.** Associations of H_2_ blocker discontinuation versus continuation with KFRT and all-cause mortality. Statistical program code for the clone-censor-weighting analysis. STROBE Checklist.

## References

[1] Kantor E.D., Rehm C.D., Haas J.S., Chan A.T., Giovannucci E.L. (2015). Trends in prescription drug use among adults in the United States from 1999–2012. JAMA.

[2] Lee H.J., Lee H., Oh S.H. (2018). Chronic kidney disease (CKD) patients are exposed to more proton pump inhibitor (PPI)s compared to non-CKD patients. PLoS One.

[3] Rotman S.R., Bishop T.F. (2013). Proton pump inhibitor use in the U.S. ambulatory setting, 2002–2009. PLoS One.

[4] Muheim L., Signorell A., Markun S. (2021). Potentially inappropriate proton-pump inhibitor prescription in the general population: a claims-based retrospective time trend analysis. Ther Adv Gastroenterol.

[5] Forgacs I., Loganayagam A. (2008). Overprescribing proton pump inhibitors. BMJ.

[6] Filion K.B., Chateau D., Targownik L.E. (2014). Proton pump inhibitors and the risk of hospitalisation for community-acquired pneumonia: replicated cohort studies with meta-analysis. Gut.

[7] Kwok C.S., Arthur A.K., Anibueze C.I., Singh S., Cavallazzi R., Loke Y.K. (2012). Risk of Clostridium difficile infection with acid suppressing drugs and antibiotics: meta-analysis. Am J Gastroenterol.

[8] Kieboom B.C.T., Kiefte-de Jong J.C., Eijgelsheim M. (2015). Proton pump inhibitors and hypomagnesemia in the general population: a population-based cohort study. Am J Kidney Dis.

[9] Blank M.L., Parkin L., Paul C., Herbison P. (2014). A nationwide nested case-control study indicates an increased risk of acute interstitial nephritis with proton pump inhibitor use. Kidney Int.

[10] Lazarus B., Chen Y., Wilson F.P. (2016). Proton pump inhibitor use and the risk of chronic kidney disease. JAMA Intern Med.

[11] Nochaiwong S., Ruengorn C., Awiphan R. (2018). The association between proton pump inhibitor use and the risk of adverse kidney outcomes: a systematic review and meta-analysis. Nephrol Dial Transplant.

[12] Pyne L., Smyth A., Molnar A.O. (2024). The effects of pantoprazole on kidney outcomes: post hoc observational analysis from the COMPASS trial: post hoc Observational analysis from the COMPASS trial. J Am Soc Nephrol.

[13] Kidney Disease: Improving Global Outcomes (KDIGO) CKD Work Group (2024). Kidney disease: improving global outcomes (KDIGO) CKD work group. KDIGO 2024 clinical practice guideline for the evaluation and management of chronic kidney disease. Kidney Int.

[14] Hernán M.A., Robins J.M. (2016). Using big data to emulate a target trial when a randomized trial is not available. Am J Epidemiol.

[15] Fu E.L. (2023). Target trial emulation to improve causal inference from observational data: what, why, and how?: what, why, and how?. J Am Soc Nephrol.

[16] Yoshida K., Solomon D.H., Kim S.C. (2015). Active-comparator design and new-user design in observational studies. Nat Rev Rheumatol.

[17] Maringe C., Benitez Majano S., Exarchakou A. (2020). Reflection on modern methods: trial emulation in the presence of immortal-time bias. Assessing the benefit of major surgery for elderly lung cancer patients using observational data. Int J Epidemiol.

[18] Zhao S.S., Lyu H., Yoshida K. (2021). Versatility of the clone-censor-weight approach: response to “trial emulation in the presence of immortal-time bias.”. Int J Epidemiol.

[19] Hattori K., Sakaguchi Y., Oka T. (2024). Estimated effect of restarting renin-angiotensin system inhibitors after discontinuation on kidney outcomes and mortality. J Am Soc Nephrol.

[20] Kawaoka T., Sakaguchi Y., Oka T. (2025). Seasonal variations in the association between proteinuria and kidney failure. Nephrol Dial Transplant.

[21] Yamaguchi S., Hamano T., Doi Y. (2020). Hidden hypocalcemia as a risk factor for cardiovascular events and all-cause mortality among patients undergoing incident hemodialysis. Sci Rep.

[22] Matsuo S., Imai E., Horio M. (2009). Revised equations for estimated GFR from serum creatinine in Japan. Am J Kidney Dis.

[23] Fu E.L., van Diepen M., Xu Y. (2021). Pharmacoepidemiology for nephrologists (part 2): Potential biases and how to overcome them. Clin Kidney J.

[24] Hernán M.A., Brumback B., Robins J.M. (2000). Marginal structural models to estimate the causal effect of zidovudine on the survival of HIV-positive men. Epidemiology.

[25] Royston P. (2004). Multiple imputation of missing values. STATA J.

[26] Xie Y., Bowe B., Li T., Xian H., Balasubramanian S., Al-Aly Z. (2016). Proton pump inhibitors and risk of incident CKD and progression to ESRD. J Am Soc Nephrol.

[27] Xie Y., Bowe B., Li T., Xian H., Yan Y., Al-Aly Z. (2017). Long-term kidney outcomes among users of proton pump inhibitors without intervening acute kidney injury. Kidney Int.

[28] Moledina D.G., Perazella M.A. (2017). Drug-induced acute interstitial nephritis. Clin J Am Soc Nephrol.

[29] Park C.H., Kim E.H., Roh Y.H., Kim H.Y., Lee S.K. (2014). The association between the use of proton pump inhibitors and the risk of hypomagnesemia: a systematic review and meta-analysis. PLoS One.

[30] Perazella M.A. (2013). Proton pump inhibitors and hypomagnesemia: a rare but serious complication. Kidney Int.

[31] Sakaguchi Y., Hamano T., Matsui I. (2019). Low magnesium diet aggravates phosphate-induced kidney injury. Nephrol Dial Transplant.

[32] Sakaguchi Y., Shoji T., Hayashi T. (2012). Hypomagnesemia in type 2 diabetic nephropathy: a novel predictor of end-stage renal disease. Diabetes Care.

[33] Tin A., Grams M.E., Maruthur N.M. (2015). Results from the Atherosclerosis Risk in Communities study suggest that low serum magnesium is associated with incident kidney disease. Kidney Int.

[34] Heerspink H.J.L., Stefánsson B.V., Correa-Rotter R. (2020). Dapagliflozin in patients with chronic kidney disease. N Engl J Med.

[35] Herrington W.G., Staplin N., The EMPA-KIDNEY Collaborative Group (2023). Empagliflozin in patients with chronic kidney disease. N Engl J Med.

[36] Bakris G.L., Agarwal R., Anker S.D. (2020). Effect of finerenone on chronic kidney disease outcomes in type 2 diabetes. N Engl J Med.

[37] Perkovic V., Tuttle K.R., Rossing P. (2024). Effects of semaglutide on chronic kidney disease in patients with type 2 diabetes. N Engl J Med.

[38] Blum M.F., Neuen B.L., Grams M.E. (2025). Risk-directed management of chronic kidney disease. Nat Rev Nephrol.

[39] Wallis K.A., Andrews A., Henderson M. (2017). Swimming against the tide: primary care physicians’ views on deprescribing in everyday practice. Ann Fam Med.

[40] Steinman M.A., Landefeld C.S. (2018). Overcoming inertia to improve medication use and deprescribing. JAMA.

[41] Oosting I.J., Colombijn J.M.T., Kaasenbrood L. (2024). Polypharmacy in patients with CKD: a systematic review and meta-analysis. Kidney360.

