# Supplementary material for: Causal Effects of Early Discontinuation of Proton Pump Inhibitors on Kidney Failure
Source: Kidney Int Rep. 2026 Jul 20;11(10):106709. doi: 10.1016/j.ekir.2026.106709 (PMC13487617; doi:10.1016/j.ekir.2026.106709)
Supplement: Supplementary file 1 [file mmc1.pdf]

Supplementary Materials

Table of Contents

Supplementary Table S1. Brief protocol of the target trial and its emulation using the OCKR database: Comparison between PPI and H_2 blocker initiation

Supplementary Table S2. Brief protocol of the target trial and its emulation using the OCKR database: Comparison between PPI discontinuation and continuation

Supplementary Table S3. Baseline characteristics of new users of PPIs and H_2 blockers at treatment initiation before and after IPTW

Supplementary Table S4. Associations of PPIs versus H_2 blockers with KFRT using a Fine-Grey subdistribution hazard model accounting for the competing risk of death

Supplementary Table S5. Distribution of the inverse probability of censoring weights in the clone-censor-weighting analysis

Supplementary Table S6. Associations of H_2 blocker discontinuation versus continuation with KFRT and all-cause mortality in on-treatment analyses

Supplementary Table S7. Associations of H_2 blocker discontinuation versus continuation with KFRT and all-cause mortality

Supplementary Figure S1. Process of the cloning, censoring, and weighting method

Supplementary Figure S2. Flow diagram of the study patients for the emulated trial comparing PPIs and H_2 blockers

Supplementary Figure S3. Balance of baseline characteristics with standardized mean difference before and after weighting

Supplementary Figure S4. Flow diagram of the study patients for the emulated trial comparing PPI discontinuation and continuation

Supplementary Figure S5. Weighted cumulative incidence curves of KFRT and all-cause mortality for PPI versus H_2 blocker

Supplementary Figure S6. Associations of PPIs versus H_2 blockers with KFRT across different subgroups of patients

Statistical program code for the clone-censor-weighting analysis

STROBE Checklist

Supplementary Table S1. Brief protocol of the target trial and its emulation using the OCKR database: Comparison between PPIs and H_2 blockers initiation

Protocol element	Target trial	Emulation with observational data from the OCKR database
Eligibility criteria	Individuals<br>1) aged 20 years or older<br>2) with CKD (eGFR 10–60 mL/min/1.73m^2)<br>3) no history of KFRT<br>4) no use of PPIs or H_2 blockers at enrollment	Same as target trial
Treatment strategies	1. Initiate PPIs only<br>2. Initiate H_2 blockers only	Same as target trial
Treatment assignment	Randomization, open-label	Eligible individuals are assigned at baseline to the treatment strategies that the data are consistent with.<br>To emulate randomization, we adjusted for the baseline confounders using IPTW.
Outcome	KFRT, defined as the initiation of maintenance hemodialysis, peritoneal dialysis, or kidney transplantation	Same as target trial
Causal estimand	Intention-to-treat effect	Same as target trial
Start and end of follow-up	Starts at randomization and ends at the occurrence of the outcome, administrative censoring, or 5 years of follow-up	Starts at medication initiation and ends at the initiation of KFRT, death, loss to follow-up, the end of the study period, or 5 years of follow-up
Statistical analysis	Cox proportional hazards models are used to estimate the treatment effects.	IPTW-weighted Cox proportional hazards models, adjusting for baseline confounders, are used to estimate the treatment effects.

Abbreviations: OCKR, Osaka Consortium for Kidney Disease Research; PPI, proton pump inhibitor; H_2 blocker, histamine$_2$ receptor antagonist; CKD, chronic kidney disease; eGFR, estimated glomerular filtration rate; KFRT, kidney failure with replacement therapy; IPTW, inverse probability of treatment weighting.

Supplementary Table S2. Brief protocol of the target trial and its emulation using the OCKR database: Comparison between PPI discontinuation and continuation

Protocol element	Target trial	Emulation with observational data from the OCKR database
Eligibility criteria	Individuals<br>1) aged 20 years or older<br>2) with CKD (eGFR 10–60 mL/min/1.73m²)<br>3) no history of KFRT<br>4) no use of PPIs at enrollment	Same as target trial
Treatment strategies	1. Discontinue PPIs within 90 days (grace period)<br>2. Continue PPIs beyond 90 days	Same as target trial
Treatment assignment	Randomization, open-label	To emulate randomization, we cloned the individuals into two identical replicates. Each replicate is assigned to one of the two treatment strategies.
Outcome	KFRT, defined as the initiation of maintenance hemodialysis, peritoneal dialysis, or kidney transplantation	Same as target trial
Causal estimand	Intention-to-treat effect	Per protocol effect: effect of adhering to the assigned strategies
Start and end of follow-up	Starts at randomization and ends at occurrence of the outcome, administrative censoring, or 5 years of follow-up	Starts at medication initiation and ends at the initiation of KFRT, death, loss to follow-up, the end of the study period, or 5 years of follow-up
Statistical analysis	Cox proportional hazards models are used to estimate the treatment effects	IPCW-weighted pooled logistic regression models, accounting for artificial censoring during the grace period, are used to estimate the treatment effects.

Abbreviations: OCKR, Osaka Consortium for Kidney Disease Research; PPI, proton pump inhibitor; CKD, chronic kidney disease; eGFR, estimated glomerular filtration rate; KFRT, kidney failure with replacement therapy; IPCW, inverse probability of censoring weighting.

Supplementary Table S3. Baseline characteristics of new users of PPIs and H_2 blockers at treatment initiation before and after IPTW

Characteristics	Missing data n (%)	Overall n = 4,614	Before IPTW PPI users n = 3,512	Before IPTW H_2 blocker users n = 1,102	Before IPTW SMD[a]	After IPTW PPI users	After IPTW H_2 blocker users	After IPTW SMD[a]
**Demographic characteristics**								
Men, n(%)	0 (0)	2,818 (61)	2,168 (62)	650 (59)	0.06	2,145 (61)	683 (61)	0.00
Age, years	0 (0)	71 (13)	72 (13)	68 (15)	0.31	71 (13)	71 (13)	0.02
Body mass index, kg/m^2	295 (6)	22.2 (4.4)	22.3 (4.3)	22.1 (4.4)	0.04	22.3 (4.3)	22.2 (4.5)	0.02
Systolic blood pressure, mmHg	589 (13)	136 (23)	137 (23)	135 (22)	0.09	136 (23)	137 (23)	0.02
Diabetes mellitus, n (%)	0 (0)	1,877 (41)	1,451 (41)	426 (39)	0.05	1,427 (41)	444 (40)	0.02
Prior cardiovascular diseases, n(%)	0 (0)	1,446 (31)	1,197 (34)	249 (23)	0.25	1,098 (31)	344 (31)	0.01
Number of hospitalizations in the preceding year, n	0 (0)	0.3 (0.7)	0.4 (0.7)	0.3 (0.9)	0.04	0.4 (0.7)	0.4 (1.0)	0.02
Serum creatinine measurements per year, n/year, median (IQR)	0 (0)	12.5 (7.7–20.6)	12.7 (8.0–20.9)	11.8 (7.0–19.8)	0.03	12.5 (7.8–20.6)	12.6 (7.3–21.2)	0.00
**Laboratory data**								
Hemoglobin, g/dL	30 (1)	11.3 (2.2)	11.2 (2.2)	11.5 (2.2)	0.15	11.3 (2.2)	11.2 (2.2)	0.02
Sodium, mEq/L	36 (1)	139 (4)	139 (4)	139 (4)	0.12	139 (4)	139 (5)	0.10
Potassium, mEq/L	34 (1)	4.4 (0.6)	4.4 (0.6)	4.4 (0.6)	0.05	4.4 (0.6)	4.4 (0.7)	0.03
Corrected calcium, mg/dL[b]	295 (6)	9.3 (0.9)	9.3 (0.8)	9.2 (1.1)	0.11	9.3 (0.8)	9.3 (1.0)	0.01
Phosphate, mg/dL	747 (16)	3.7 (0.9)	3.7 (1.0)	3.7 (0.9)	0.01	3.7 (1.0)	3.7 (0.9)	0.01
Magnesium, mg/dL	2,037 (44)	2.1 (0.4)	2.1 (0.4)	2.1 (0.5)	0.03	2.1 (0.4)	2.1 (0.5)	0.02
Albumin, g/dL	64 (1)	3.5 (0.7)	3.5 (0.7)	3.6 (0.7)	0.17	3.5 (0.7)	3.5 (0.7)	0.03

Supplementary Table S3. Baseline characteristics of new users of PPIs and H_2 blockers at treatment initiation before and after IPTW (continued)

eGFR, ml/min/1.73m^2	0 (0)	30 (17)	30 (17)	32 (17)	0.12	30 (17)	30 (17)	0.00
Total cholesterol, mg/dL	1,023 (22)	193 (63)	191 (66)	197 (54)	0.10	193 (66)	193 (53)	0.00
Serum urate, mg/dL	289 (6)	6.7 (2.0)	6.7 (2.0)	6.9 (1.9)	0.10	6.7 (2.0)	6.7 (1.9)	0.01
C-reactive protein, mg/dL, median (IQR)	228 (5)	0.2 (0.1–0.9)	0.2 (0.1–1.0)	0.1 (0.1–0.7)	0.06	0.2 (0.1–0.9)	0.2 (0.1–1.0)	0.04
UPCR, g/gCre, median (IQR)	682 (15)	0.9 (0.2–3.2)	0.9 (0.2–3.3)	0.8 (0.2–2.9)	0.07	0.9 (0.2–3.2)	0.9 (0.2–3.1)	0.03
**Prescriptions**								
ACEIs/ARBs, n (%)	0 (0)	1,916 (42)	1,421 (40)	495 (45)	0.09	1,455 (41)	449 (40)	0.02
Calcium channel blockers, n (%)	0 (0)	2,335 (51)	1,802 (51)	533 (48)	0.06	1,778 (51)	566 (51)	0.00
Beta blockers, n (%)	0 (0)	1,154 (25)	928 (26)	226 (21)	0.14	876 (25)	278 (25)	0.00
Loop diuretics, n (%)	0 (0)	1,704 (37)	1,358 (39)	346 (31)	0.15	1,298 (37)	419 (38)	0.01
Thiazide diuretics, n (%)	0 (0)	458 (10)	358 (10)	100 (9)	0.04	350 (10)	120 (11)	0.03
MRAs, n (%)	0 (0)	487 (11)	407 (12)	80 (7)	0.14	372 (11)	119 (11)	0.00
Systemic steroids, n (%)	0 (0)	1,104 (24)	784 (22)	320 (29)	0.16	844 (24)	283 (25)	0.03
NSAIDs, n(%)	0 (0)	118 (3)	90 (3)	28 (3)	0.00	90 (3)	28 (3)	0.00
Antithrombotics, n(%)	0 (0)	1,178 (26)	1,012 (29)	166 (15)	0.32	897 (26)	294 (26)	0.02

Abbreviations: PPI, proton pump inhibitor; H_2 blocker, histamine$_2$ receptor antagonist; IPTW, inverse probability of treatment weight; SMD, standardized mean difference; eGFR, estimated glomerular filtration rate; IQR, interquartile range; UPCR, urinary protein-creatinine ratio; ACEI, angiotensin-converting enzyme inhibitor; ARB, angiotensin II receptor blocker; MRA, mineralocorticoid receptor antagonist; NSAIDs, nonsteroidal anti-inflammatory drugs.

Data are presented as the mean (standard deviation) unless otherwise indicated.

[a] An SMD of ≥ 0.10 indicates a nonnegligible imbalance between the groups.

[b] Serum calcium was corrected for serum albumin levels using the formula: corrected calcium (mg/dL) = total calcium + 0.8 × (4.0 − albumin), if albumin <4.0 (g/dL).

Supplementary Table S3. Baseline characteristics of new users of PPIs and H_2 blockers at treatment initiation before and after IPTW (continued)

SI conversion factors: To convert hemoglobin, albumin, and C-reactive protein to g/L, multiply by 10; sodium and potassium to mmol/L, multiply by 1.0; and calcium to mmol/L, multiply by 0.25; phosphate to mmol/L, multiple by 0.323; magnesium to mmol/L, multiple by 0.4114; total cholesterol to mmol/L, multiple by 0.0259; urate to mmol/L, multiple by 0.0595.

Supplementary Table S4. Associations of PPIs versus H2 blockers with KFRT using a Fine-Grey subdistribution hazard model accounting for the competing risk of death

Outcomes	Treatment strategies	No. of Events	Unweighted sHR (95% CI)	Weighted sHR (95% CI)[a]
KFRT	H_2 blockers	145	Reference	Reference
	PPIs	509	1.37 (1.14–1.64)	1.34 (1.10–1.64)

Abbreviations: PPI, proton pump inhibitor; H_2 blocker, $histamine_2$ receptor antagonist; KFRT, kidney failure with replacement therapy; CI, confidence interval; sHR, subdistribution hazard ratio.

[a] Weighted subdistribution hazard ratios were estimated using a Fine-Gray subdistribution hazard model accounting for the competing risk of death, with the inverse probability of treatment weights derived from the following covariates: sex, age, body mass index, systolic blood pressure, history of diabetes mellitus, history of cardiovascular disease, the number of hospitalizations in the preceding year, the annual number of serum creatinine measurements, hemoglobin, sodium, potassium, corrected calcium, phosphate, magnesium, serum urate, albumin, total cholesterol, estimated glomerular filtration rate, log-transformed C-reactive protein, log-transformed urinary protein-creatinine ratio, prescriptions of angiotensin-converting enzyme inhibitors/angiotensin II receptor blockers, calcium channel blockers, beta-blockers, loop diuretics, thiazide diuretics, and mineralocorticoid receptor antagonists, nonsteroidal anti-inflammatory drugs, steroids, antithrombotics.

Supplementary Table S5. Distribution of the inverse probability of censoring weights in the clone-censor-weighting analysis

Outcome	Treatment strategies	Min	Mean (SD)	99th percentile	Max
KFRT	Continuation	0.24	0.99 (0.11)	1.42	4.61
	Discontinuation	0.18	1.02 (0.55)	3.29	7.83

Abbreviations: KFRT, kidney failure with replacement therapy; SD, standard deviation.

Supplementary Table S6. Associations of PPI discontinuation versus continuation with KFRT and all-cause mortality in on-treatment analyses

Outcomes	Treatment strategies	No. of Events in the emulated Trial	5-year Absolute Risk, % (95% CI)	5-year Risk Difference, % (95% CI)	Hazard Ratio (95% CI)
KFRT	Continuation	290	22.0 (19.7 to 24.4)	Reference	Reference
	Discontinuation	112	15.4 (12.8 to 18.2)	-6.7 (-9.8 to -3.8)	0.67 (0.55 to 0.80)
All-cause mortality	Continuation	367	23.9 (21.8 to 26.5)	Reference	Reference
	Discontinuation	264	22.0 (19.5 to 24.9)	-1.9 (-4.2 to 0.4)	0.91 (0.81 to 1.02)

Abbreviations: PPI, proton pump inhibitor; KFRT, kidney failure with replacement therapy; CI, confidence interval; HR, hazard ratio.

Analyses were adjusted through the inverse probability of censoring weights derived from the following covariates: sex, age, body mass index, systolic blood pressure, history of diabetes mellitus, history of cardiovascular disease, the number of hospitalizations in the preceding year, the annual number of serum creatinine measurements, hemoglobin, sodium, potassium, corrected calcium, phosphate, magnesium, serum urate, albumin, total cholesterol, estimated glomerular filtration rate, log-transformed C-reactive protein, log-transformed urinary protein-creatinine ratio, prescriptions of angiotensin-converting enzyme inhibitors/angiotensin II receptor blockers, calcium channel blockers, beta-blockers, loop diuretics, thiazide diuretics, and mineralocorticoid receptor antagonists, nonsteroidal anti-inflammatory drugs, steroids, antithrombotics.

Supplementary Table S7. Associations of H_2 blocker discontinuation versus continuation with KFRT and all-cause mortality

Outcomes	Treatment strategies	No. of Events in the emulated Trial	5-year Absolute Risk, % (95% CI)	5-year Risk Difference, % (95% CI)	Hazard Ratio (95% CI)
KFRT	Continuation	99	15.0 (12.8 to 17.9)	Reference	Reference
	Discontinuation	53	15.3 (11.3 to 19.2)	0.3 (-3.4 to 3.8)	1.02 (0.76 to 1.30)
All-cause mortality	Continuation	183	22.9 (20.5 to 25.6)	Reference	Reference
	Discontinuation	114	21.3 (18.2 to 24.8)	-1.5 (-5.2 to 2.1)	0.92 (0.75 to 1.11)

Abbreviations: H_2 blocker, histamine$_2$ receptor antagonist; KFRT, kidney failure with replacement therapy; CI, confidence interval.

Analyses were adjusted through the inverse probability of censoring weights derived from the following covariates: sex, age, body mass index, systolic blood pressure, history of diabetes mellitus, history of cardiovascular disease, the number of hospitalizations in the preceding year, the annual number of serum creatinine measurements, hemoglobin, sodium, potassium, corrected calcium, phosphate, magnesium, serum urate, albumin, total cholesterol, estimated glomerular filtration rate, log-transformed C-reactive protein, log-transformed urinary protein-creatinine ratio, prescriptions of angiotensin-converting enzyme inhibitors/angiotensin II receptor blockers, calcium channel blockers, beta-blockers, loop diuretics, thiazide diuretics, and mineralocorticoid receptor antagonists, nonsteroidal anti-inflammatory drugs, steroids, antithrombotics.

Supplementary Figure S1. Process of the cloning, censoring, and weighting method

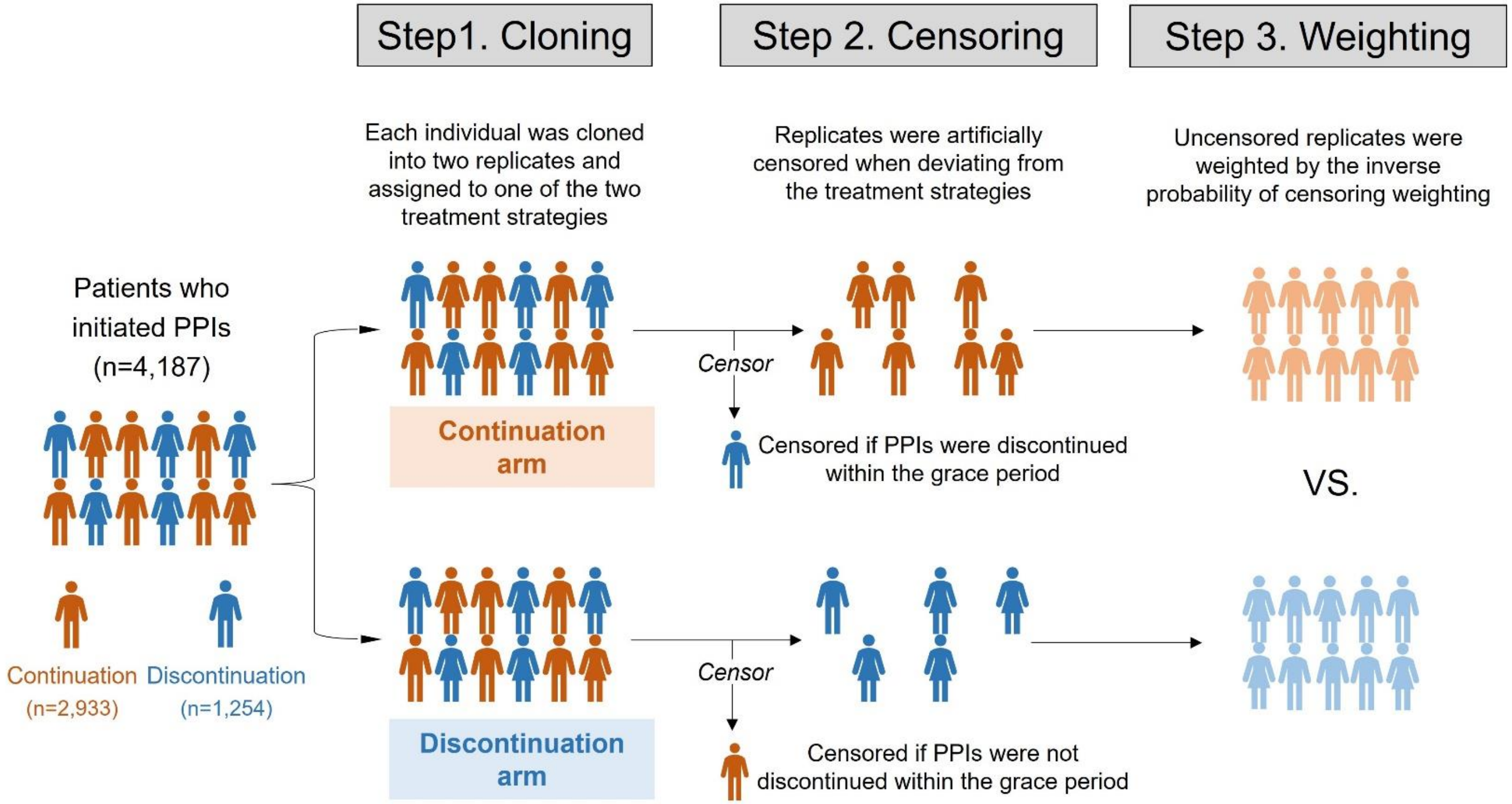

The clone-censor-weighting approach was used to emulate the target trial in this study. First, each individual was cloned into two identical replicates and assigned to one of the two treatment strategies. Since these two groups comprised exactly the same replicates, baseline confounding cannot arise. Second, replicates were artificially censored when they deviated from the assigned treatment strategies. Specifically, those in the continuation arm were censored if PPIs were discontinued within the grace period, whereas those in the discontinuation arm were censored if PPIs were not discontinued within the grace period. Finally, the IPCW was used to deal with the potential selection bias induced by artificial censoring.

Abbreviations: PPI, proton pump inhibitor; IPCW, inverse probability of censoring weighting.

Supplementary Figure S2. Flow diagram of the study patients for the emulated trial comparing PPIs and H_2 blockers

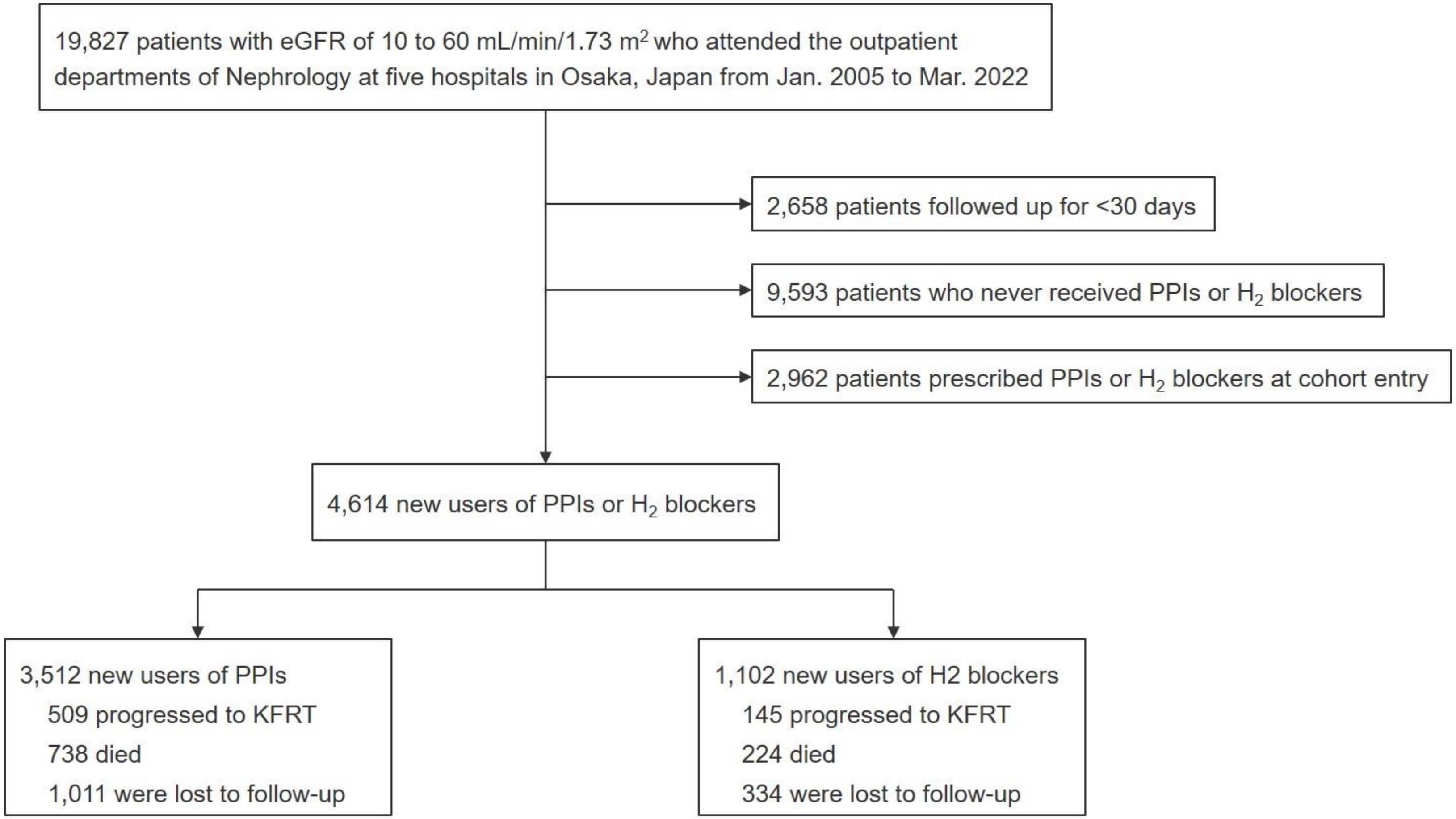

Abbreviations: PPI, proton pump inhibitor; H_2 blocker, histamine$_2$ receptor antagonist; eGFR, estimated glomerular filtration rate; KFRT, kidney failure with replacement therapy.

Supplementary Figure S3. Balance of baseline characteristics with standardized mean difference before and after weighting

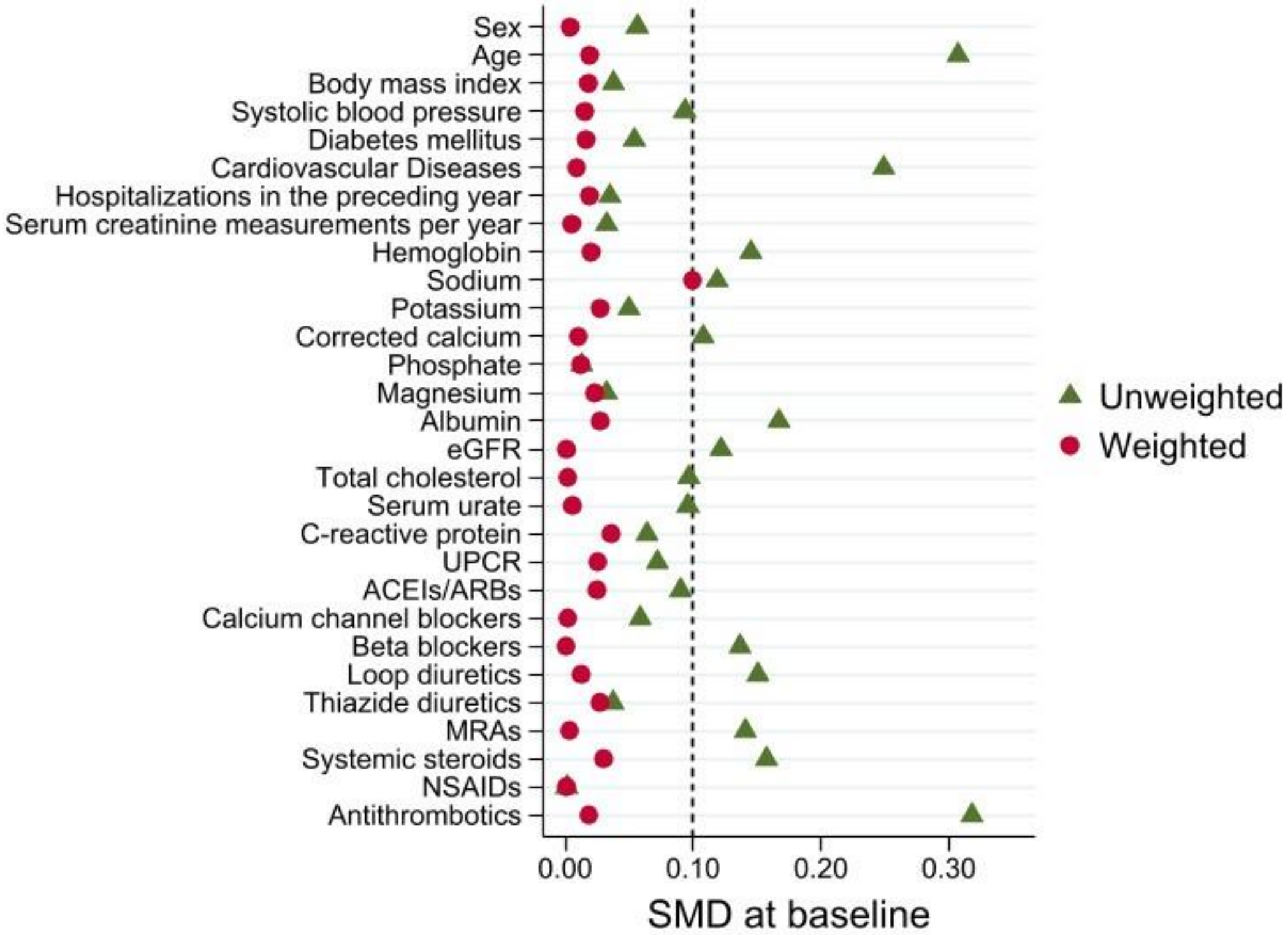

An SMD of ≥ 0.10 indicates a nonnegligible imbalance between the groups.

Abbreviations: eGFR, estimated glomerular filtration rate; UPCR, urinary protein-creatinine ratio; ACEI, angiotensin-converting enzyme inhibitor; ARB, angiotensin II receptor blocker; MRA, mineralocorticoid receptor antagonist; NSAIDs, nonsteroidal anti-inflammatory drugs; SMD, standardized mean difference.

Supplementary Figure S4. Flow diagram of the study patients for the emulated trial comparing PPI discontinuation and continuation

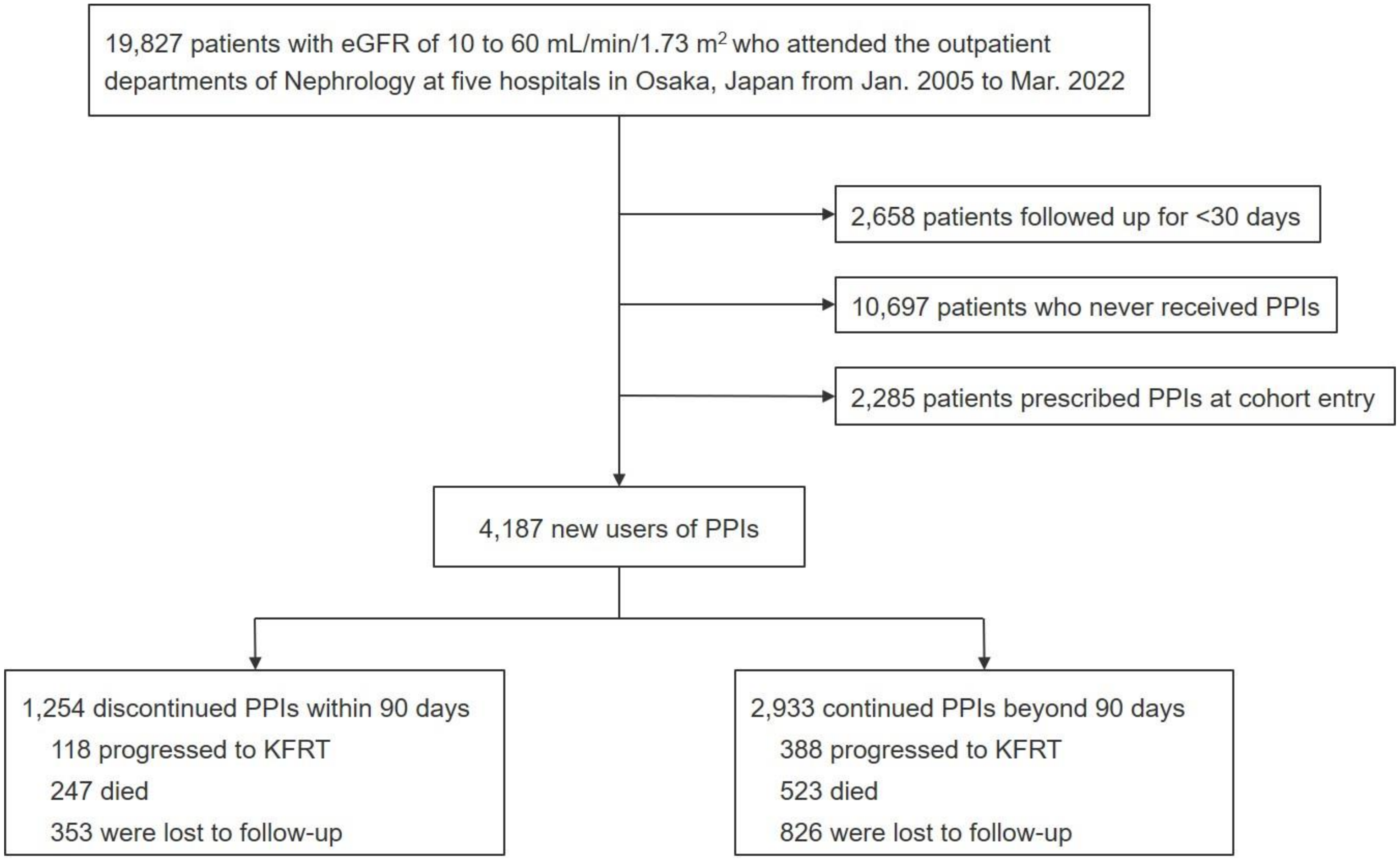

Abbreviations: PPI, proton pump inhibitor; eGFR, estimated glomerular filtration rate; KFRT, kidney failure with replacement therapy.

Supplementary Figure S5. Weighted cumulative incidence curves of KFRT and all-cause mortality for PPIs versus H_2 blockers

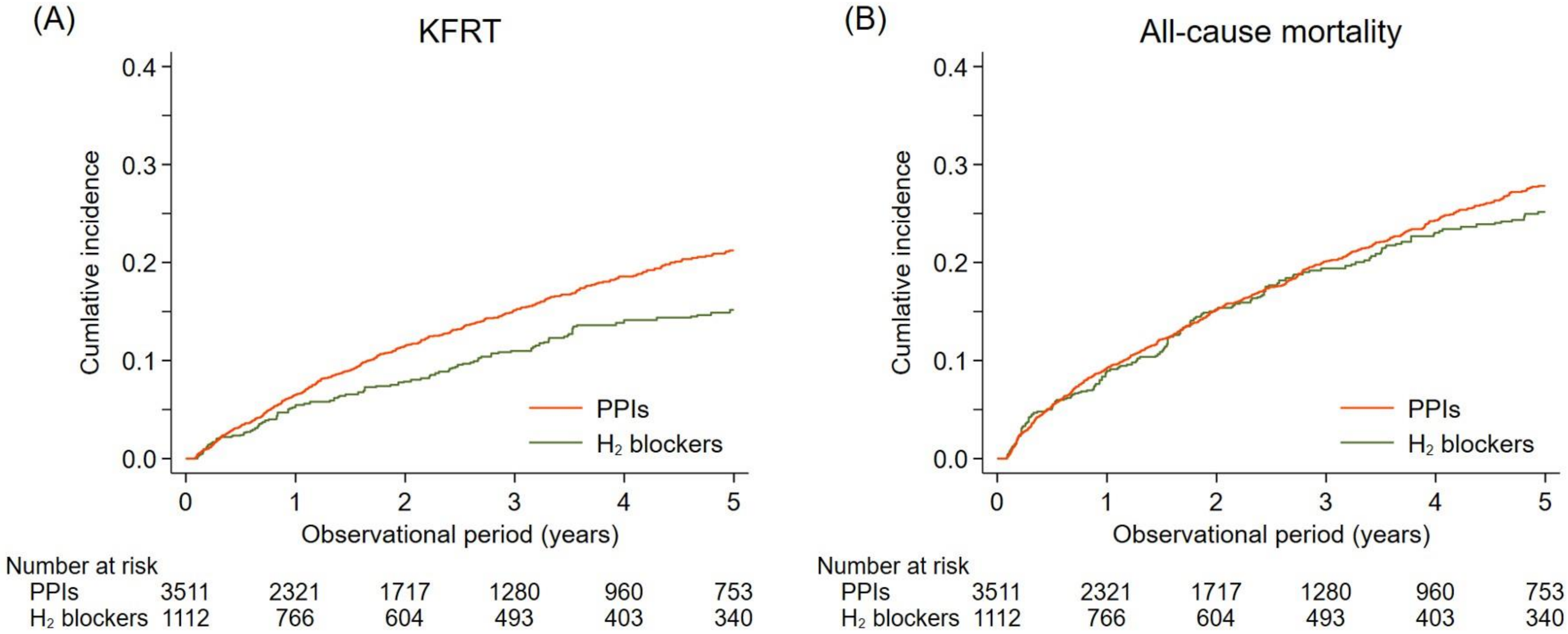

The median length of follow-up was 2.1 years (interquartile range, 0.7–4.8 years). The cumulative incidence of KFRT was significantly higher in the PPI group than in the H_2 blocker group (panel A; $p = 0.001$). The cumulative incidence of all-cause mortality did not significantly differ between the groups (panel B; $p = 0.10$).

Abbreviations: KFRT, kidney failure with replacement therapy; PPI, proton pump inhibitor; H_2 blocker, $histamine_2$ receptor antagonist.

Supplementary Figure S6. Associations of PPIs versus H_2 blockers with KFRT across different subgroups of patients

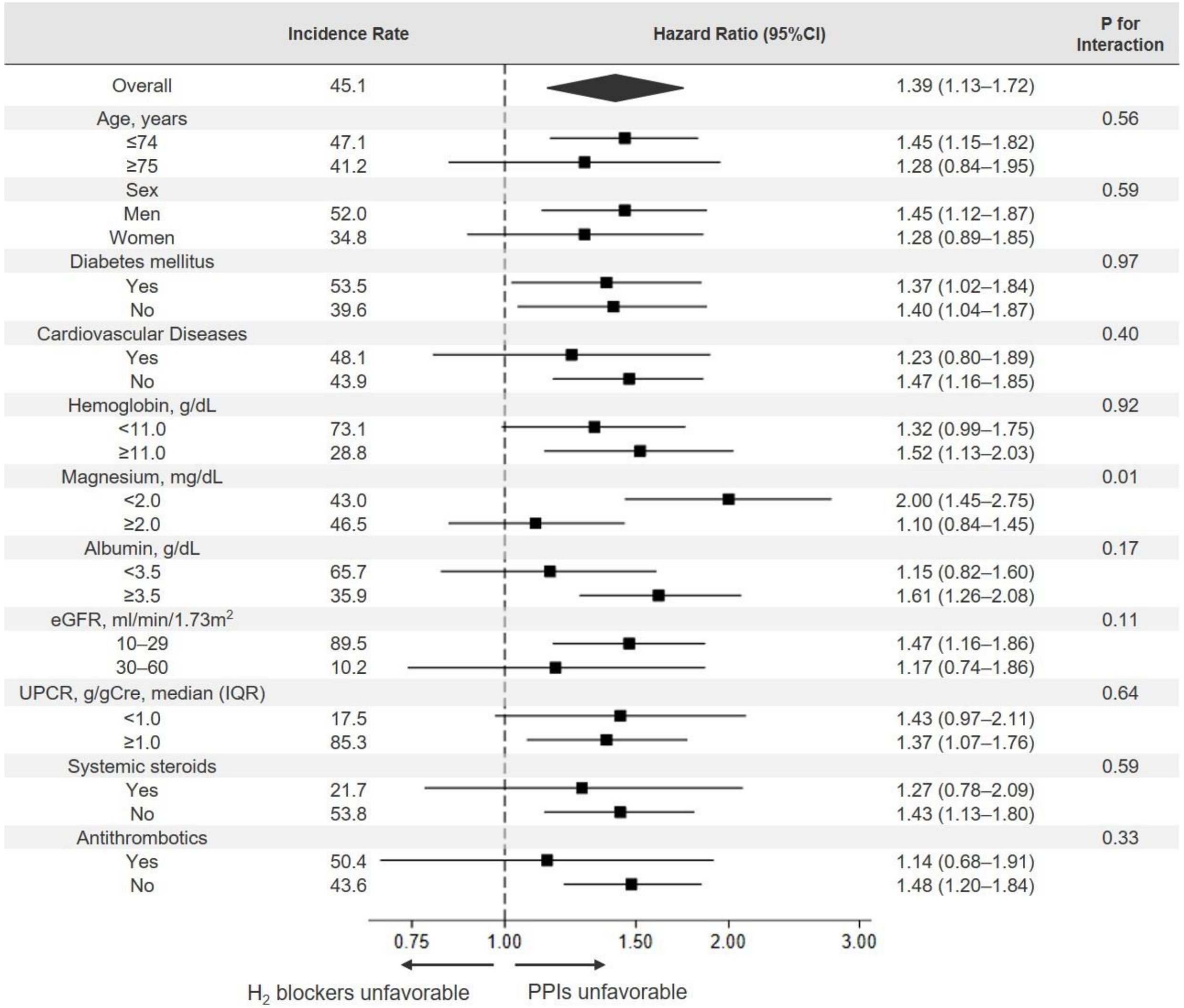

Supplementary Figure S6. Associations of PPIs versus H_2 blockers with KFRT across different subgroups of patients (continued)

The dots represent hazard ratios for kidney failure with replacement therapy in PPI discontinuation versus continuation. The error bars represent the 95% confidence intervals. Incidence rates were expressed as the number of events per 1,000 person-years. Hazard ratios were estimated using the inverse probability of treatment weights derived from the following covariates: sex, age, body mass index, systolic blood pressure, history of diabetes mellitus, history of cardiovascular disease, the number of hospitalizations in the preceding year, the annual number of serum creatinine measurements, hemoglobin, sodium, potassium, corrected calcium, phosphate, magnesium, serum urate, albumin, total cholesterol, estimated glomerular filtration rate, log-transformed C-reactive protein, log-transformed urinary protein-creatinine ratio, prescriptions of angiotensin-converting enzyme inhibitors/angiotensin II receptor blockers, calcium channel blockers, beta-blockers, loop diuretics, thiazide diuretics, and mineralocorticoid receptor antagonists, nonsteroidal anti-inflammatory drugs, steroids, antithrombotics.

Abbreviations: PPI, proton pump inhibitor; H_2 blocker, histamine H_2 receptor antagonist; KFRT, kidney failure with replacement therapy; eGFR, estimated glomerular filtration rate; UPCR, urinary protein-creatinine ratio.

Statistical program code for the clone-censor-weighting analysis

The following Stata code implements the clone-censor-weighting analysis used to estimate the effect of PPI discontinuation versus continuation. The analysis was performed using a person-time (long-format) dataset, in which each row represents one patient-day interval of follow-up. The program takes two arguments: group, indicating the observed treatment status, and grace, specifying the length of the grace period in days.

Dataset variables and program arguments

Argument / Variable	Description
`id`	Patient identifier
`time`	Follow-up time in days from PPI initiation (time 0 = day of initiation)
`time_end`	End of each person-time interval
`time_max`	Last observed time point for each replicate
`followtime`	Total follow-up time until the outcome, censoring, or end of follow-up
`RRT`	Indicator for kidney failure with replacement therapy (1 = event)
`DEATH`	Indicator for all-cause mortality (1 = event)
`CENS`	Indicator for administrative censoring (loss to follow-up or end of study)
`ppi_end_time`	Time at which PPI was discontinued
`arm`	Assigned treatment arm after cloning (0 = Continuation, 1 = Discontinuation)
`clone_id`	Unique identifier for each cloned replicate
`ACENS`	Indicator for artificial censoring at the time of protocol deviation
`$base`	Baseline covariates measured at PPI initiation
`$tvc`	Time-varying covariates updated during follow-up
`group (argument)`	Observed treatment status passed to the program: 1 = patient discontinued PPI within the grace period, 0 = continued
`grace (argument)`	Length of the grace period in days (90 in the primary analysis)

Program code

```
*--- CCW program ---
program define ccw
syntax , group(name max=1) grace(integer)

*=== Step 1. Cloning ===
* Duplicate each patient into two replicates and assign to each arm
expand 2
bysort id time: gen arm = _n-1
label define arm_lab 0 "Continuation" 1 "Discontinuation"
label value arm arm_lab
egen clone_id = group(id)
replace clone_id = cond(arm==0, clone_id*10, clone_id*10+1)



*=== Step 2. Censoring ===
* Continuation arm: patients who discontinued PPI deviate from the strategy.
* Their follow-up is truncated at the time of PPI discontinuation.
replace RRT=0 if arm == 0 & `group'==1
replace DEATH=0 if arm == 0 & `group'==1
replace followtime = ppi_end_time if arm == 0 & `group'==1

* Discontinuation arm: patients who did not discontinue PPI within the grace period
deviate from the strategy; follow-up is truncated at the grace period.
replace RRT=0 if arm == 1 & `group'==0 & followtime>`grace'
replace DEATH=0 if arm == 1 & `group'==0 & followtime>`grace'
replace followtime = `grace' if arm == 1 & `group'==0 & followtime>`grace'

* Define artificial censoring (ACENS) at the time of protocol deviation
gen ACENS = 0
replace ACENS = 1 if arm==0 & `group'==1 & time==ppi_end_time & (RRT!=1 & DEATH!=1)
replace ACENS = 1 if arm==1 & `group'==0 & CENS!=1 & ///
  (time==time_max & time<`grace' | time==`grace') & (RRT!=1 & DEATH!=1)

* Adjust the observation rows according to the truncated follow-up time
sort clone_id time
drop if time > followtime
drop time_max
```

```
by clone_id: egen time_max=max(time)
by clone_id: replace time_end = cond(time==time_max, followtime, time[_n+1])
replace RRT=. if ACENS==1
replace DEATH=. if ACENS==1


*=== Step 3. Weighting (inverse probability of censoring weights) ===
* Denominator model: probability of remaining uncensored given baseline and time-
varying covariates, estimated separately for each arm
logistic ACENS $tvc $base c.time##c.time if arm==0 & time<=`grace',
cluster(clone_id)
predict p_cens_cont if arm==0 & time<=`grace'
logistic ACENS $tvc $base c.time##c.time if arm==1 & time<=`grace',
cluster(clone_id)
predict p_cens_discont if arm==1 & time<=`grace'
gen cens_denom = cond(ACENS==0, 1-p_cens_cont, p_cens_cont) if arm==0
replace cens_denom = cond(ACENS==0, 1-p_cens_discont, p_cens_discont) if arm==1
replace cens_denom = 1 if cens_denom==. | time==0
bysort clone_id (time): replace cens_denom = cens_denom*cens_denom[_n-1] if _n!=1
drop p_cens*

* Numerator model: probability of remaining uncensored given baseline covariates
only, used for stabilization
logistic ACENS $base c.time##c.time if arm==0 & time<=`grace', cluster(clone_id)
predict p_cens_cont if arm==0 & time<=`grace'
logistic ACENS $base c.time##c.time if arm==1 & time<=`grace', cluster(clone_id)
predict p_cens_discont if arm==1 & time<=`grace'
gen cens_num = cond(ACENS==0, 1-p_cens_cont, p_cens_cont) if arm==0
replace cens_num = cond(ACENS==0, 1-p_cens_discont, p_cens_discont) if arm==1
replace cens_num = 1 if cens_num==. | time==0
bysort clone_id (time): replace cens_num = cens_num*cens_num[_n-1] if _n!=1
drop p_cens*

* Stabilized inverse probability of censoring weights
gen stabw_acens = cens_num/cens_denom

end
```

```
*=== Outcome model ===
* IPCW-weighted pooled logistic regression with robust variance
logistic `outcome' arm c.time##c.time [pw=stabw_acens], cluster(clone_id)
```

The STROBE reporting checklist

	Item Description	Location (or reason for not reporting)
**Title and abstract**		
1a. Indicate the study's design	Indicate the study's design with a commonly used term in the title or the abstract.	Abstract
1b. Abstract	Provide in the abstract an informative and balanced summary of what was done and what was found.	Abstract
**Introduction**		
2. Background / rationale	Explain the scientific background and rationale for the investigation being reported.	Introduction; paragraphs 1 & 2
3. Objectives	State specific objectives, including any prespecified hypotheses.	Introduction; paragraph 3
**Methods**		
4. Study design	Present key elements of study design early in the paper.	Methods, Study Design and Target Trial Emulation; paragraph 1
5. Setting	Describe the setting, locations, and relevant dates, including periods of recruitment, exposure, follow-up, and data collection.	Methods, Data Collection and Study population; paragraph 1
6a. Eligibility criteria	**Cohort study:** Give the eligibility criteria, and the sources and methods of selection of participants. Describe methods of follow-up. **Case-control study:** Give the eligibility criteria, and the sources and methods of case ascertainment and control selection. Give the rationale for the choice of cases and controls. **Cross-sectional study:** Give the eligibility criteria, and the sources and methods of selection of participants.	Methods, Data Collection and Study population; paragraph 2
6b. Matching criteria	**Cohort study:** For matched studies, give matching criteria and number of exposed and unexposed. **Case-control study:** For matched studies, give matching criteria and the number of controls per case.	Not applicable
7. Variables	Clearly define all outcomes, exposures, predictors, potential confounders, and effect modifiers. Give diagnostic criteria, if applicable.	Methods, Treatment assignment; paragraphs 1 & 2<br>Methods, Study Outcome and Follow-up; paragraph 1

		Methods, Covariates; paragraphs 1 & 2
8. Data sources / measurement	For each variable of interest give sources of data and details of methods of assessment (measurement). Describe comparability of assessment methods if there is more than one group.	Methods, Data Collection and Study population; paragraph 1
9. Bias	Describe any efforts to address potential sources of bias.	Methods, Statistical Analysis, Active comparator new user design; paragraph 1<br>Methods, Statistical Analysis, Clone-censor-weighting; paragraphs 1 & 2
10. Study size	Explain how the study size was arrived at.	No formal sample size calculation
11. Quantitative variables	Explain how quantitative variables were handled in the analyses. If applicable, describe which groupings were chosen, and why.	Not applicable
12a. Statistical methods	Describe all statistical methods, including those used to control for confounding.	Methods, Statistical Analysis, Active comparator new user design; paragraph 1<br>Methods, Statistical Analysis, Clone-censor-weighting; paragraphs 1 & 2
12b. Statistical methods – subgroups and interactions	Describe any methods used to examine subgroups and interactions.	Methods, Statistical Analysis, Subgroup and Sensitivity Analyses; paragraph 1
12c. Statistical methods – missing data	Explain how missing data were addressed.	Methods, Statistical Analysis, Imputation for missing data; paragraph 1
12di. Statistical methods – loss to follow-up	**Cohort study:** If applicable, describe how loss to follow-up was addressed.	Methods, Study Outcome and Follow-up; paragraph 2
12dii. Statistical methods – matching cases and controls	**Case-control study:** If applicable, explain how matching of cases and controls was addressed.	Not applicable
12diii. Statistical methods – sampling strategy	**Cross-sectional study:** If applicable, describe analytical methods taking account of sampling strategy.	Not applicable
12e. Statistical methods – sensitivity analyses	Describe any sensitivity analyses.	Methods, Statistical Analysis, Subgroup and Sensitivity Analyses; paragraph 2
**Results**		

13a. Participant numbers	Report the numbers of individuals at each stage of the study—e.g., numbers potentially eligible, examined for eligibility, confirmed eligible, included in the study, completing follow-up, and analysed; Consider use of a flow diagram.	Results, Study Population; paragraphs 1 & 2<br>Supplementary Figure S2 & S4
13b. Participants – non-participation	Give reasons for non-participation at each stage.	Supplementary Figure S2 & S4
13c. Participants – flow diagram	Consider use of a flow diagram.	Supplementary Figure S2 & S4
14a. Descriptive data – participant characteristics	Give characteristics of study participants (e.g., demographic, clinical, social) and information on exposures and potential confounders. Present the information in a table.	Results, Study Population; paragraphs 1 & 2<br>Supplementary Table S3 & Table 1
14b. Descriptive data – missing data	Indicate the number of participants with missing data for each variable of interest.	Supplementary Table S3 & Table 1
14c. Descriptive data – follow-up time	**Cohort study:** Summarise follow-up time—e.g., average and total amount.	Results, Comparison of Clinical Outcomes between New Users of PPIs and H_2 blockers; paragraph 1<br>Results, Causal Effects of PPI Discontinuation on Clinical Outcomes; paragraph 1
15. Outcome data	**Cohort study:** Report numbers of outcome events or summary measures over time. **Case-control study:** Report numbers in each exposure category, or summary measures of exposure. **Cross-sectional study:** Report numbers of outcome events or summary measures.	Results, Comparison of Clinical Outcomes between New Users of PPIs and H_2 blockers; paragraph 1<br>Results, Causal Effects of PPI Discontinuation on Clinical Outcomes; paragraph 1
16a. Main results	Give unadjusted estimates and, if applicable, confounder-adjusted estimates and their precision (e.g., 95% confidence intervals). Make clear which confounders were adjusted for and why they were included.	Results, Comparison of Clinical Outcomes between New Users of PPIs and H_2 blockers; paragraphs 1<br>Results, Causal Effects of PPI Discontinuation on Clinical Outcomes; paragraph 2<br>Table 2 & 3
16b. Main results – category boundaries	Report category boundaries when continuous variables were categorized.	Results, Causal Effects of PPI Discontinuation on Clinical Outcomes; paragraph 3<br>Figure 2
16c. Main results – risk	If relevant, consider translating estimates of relative risk into absolute risk for a meaningful time period.	Results, Causal Effects of PPI Discontinuation on Clinical Outcomes; paragraph 2<br>Table 3

17. Other analyses	Report other analyses done—e.g., analyses of subgroups and interactions, and sensitivity analyses.	Results, Comparison of Clinical Outcomes between New Users of PPIs and H_2 blockers; paragraphs 2<br>Results, Causal Effects of PPI Discontinuation on Clinical Outcomes; paragraph 3<br>Supplementary Table S6 &S7<br>Figure 3<br>Supplementary Figure S6
**Discussion**		
18. Key results	Summarise key results with reference to study objectives.	Discussion; paragraph 1
19. Limitations	Discuss limitations of the study, taking into account sources of potential bias or imprecision. Discuss both direction and magnitude of any potential bias.	Discussion; paragraphs 6
20. Interpretation	Give a cautious overall interpretation considering objectives, limitations, multiplicity of analyses, results from similar studies, and other relevant evidence.	Discussion; paragraphs 2–5
21. Generalisability	Discuss the generalisability (external validity) of the study results.	Discussion; paragraph 6
**Other information**		
22. Funding	Give the source of funding and the role of the funders for the present study and, if applicable, for the original study on which the present article is based.	None
